# Identification of protein changes in the blood plasma of lung cancer patients subjected to chemotherapy using a 2D-DIGE approach

**DOI:** 10.1371/journal.pone.0223840

**Published:** 2019-10-17

**Authors:** Andrzej Ciereszko, Mariola A. Dietrich, Mariola Słowińska, Joanna Nynca, Michał Ciborowski, Joanna Kisluk, Anna Michalska-Falkowska, Joanna Reszec, Ewa Sierko, Jacek Nikliński

**Affiliations:** 1 Department of Gamete and Embryo Biology, Institute of Animal Reproduction and Food Research, Polish Academy of Sciences, Olsztyn, Poland; 2 Clinical Research Centre, Medical University of Białystok, Białystok, Poland; 3 Department of Clinical Molecular Biology, Medical University of Bialystok, Bialystok, Poland; 4 Department of Medical Pathomorphology, Medical University of Bialystok, Bialystok, Poland; 5 Department of Oncology, Medical University of Bialystok, Bialystok, Poland; CHA University, REPUBLIC OF KOREA

## Abstract

A comparative analysis of blood samples (depleted of albumin and IgG) obtained from lung cancer patients before chemotherapy versus after a second cycle of chemotherapy was performed using two-dimensional difference gel electrophoresis (2D-DIGE). The control group consisted of eight patients with non-cancerous lung diseases, and the experimental group consisted of four adenocarcinoma (ADC) and four squamous cell carcinoma (SCC) patients. Analyses of gels revealed significant changes in proteins and/or their proteoforms between control patients and lung cancer patients, both before and after a second cycle of chemotherapy. Most of these proteins were related to inflammation, including acute phase proteins (APPs) such as forms of haptoglobin and transferrin, complement component C3, and clusterin. The variable expression of APPs can potentially be used for profiling lung cancer. The greatest changes observed after chemotherapy were in transferrin and serotransferrin, which likely reflect disturbances in iron turnover after chemotherapy-induced anaemia. Significant changes in plasma proteins between ADC and SCC patients were also revealed, suggesting use of plasma vitronectin as a potential marker of SCC.

## Introduction

Lung cancer is the most common cancer in the world and responsible for most cancer-related mortality worldwide [1]. Symptoms of lung cancer are usually very difficult to recognise until the disease is in an advanced, non-curable state. It is estimated that only 16% of all patients will survive five or more years after diagnosis. Late diagnosis is a significant factor contributing to poor lung cancer prognosis [1]. For this reason, the development of biomarkers for effective prognosis is of utmost importance [2].

Biomarkers are biological compounds that can be used to distinguish a pathological from a normal status. Several biomolecules are used as potential cancer biomarkers, such as DNA, methylated DNA, RNA, miRNA, low molecular weight metabolites, xenobiotics, and proteins [3–5]. For example, circulating miRNAs, which are short, noncoding RNA molecules, can bind and interfere with mRNAs that are important for tumour expression pathways, and are also used for the detection of lung cancer [6]. Long, noncoding RNAs are also involved in tumourigenesis [7]. At the molecular level, proteins represent the most important functional unit that is directly responsible for a phenotype. For this reason, almost all Food and Drug Administration (FDA) approved cancer biomarkers are proteins [1,8].

There are several potential noninvasive and convenient sources for biomarker identification, such as body fluids, including serum or plasma, urine, sputum, tears, pleural effusion, and volatile organic compounds in exhaled breath concentrate [9]. Blood plasma is a convenient, noninvasive, inexpensive, and clinically relevant source of substances that can be screened for potential biomarkers. So far, several serum biomarkers for lung cancer have been identified, including fragments of cytokeratin 19 CYFRA 21–1, carcinoembryonic antigen (CEA), squamous cell carcinoma antigen (SCC-Ag), stem cell factor (SCF), neuron-specific enolase (NSE), progastrin-releasing peptide (ProGRP), epidermal growth factor receptor (EGFR), and vascular endothelial growth factor (VEGF) [1,8]. Unfortunately, the performance of individual markers has been disappointing due to their low sensitivity and specificity [1]. Therefore, it is unlikely that a single biomarker for any particular form of cancer can be identified; rather, a multi-marker approach is recommended [10–11]. For this reason, a search for new potential biomarkers is highly justified in order to identify potential candidates for incorporation into multi-marker algorithm biomarkers [12].

Two-dimensional difference gel electrophoresis (2D-DIGE) is variation of two-dimensional electrophoresis (2DE), and has found to be the preferred method for proteomic analysis of lung cancer due to its high reproducibility, sensitivity, comprehensiveness, and high throughput [13–14]. In 2D-DIGE, samples are labelled with distinct fluorescent dyes before electrophoretic separation; the two main approaches include minimal labelling and saturation labelling [15]. The former is a highly sensitive technique that employs fluorophores (Cy5, Cy3 and Cy2) with affinity to primary amino groups, covering only a small fraction (~3%) of each protein [16]. The use of three fluorophores enables electrophoretic co-separation of the sample and control, thereby greatly reducing spot matching errors within one replicate (one gel), and facilitates the creation of an internal pooled standard prepared from the mixture of all specimens used in the analysis. Saturation labelling was introduced by Kondo *et al*. [17] and is based on the complete labelling of sulfhydryl residues of cysteines using the Cy3 and Cy5 dyes. Due to its high sensitivity, saturation labelling is especially useful when sample amounts are limited, such as when using microdissected tissues [17]. However, only two fluorophores are currently available for this method, and therefore two gels must be run in order to maintain the internal standard concept [18]. For most 2D-DIGE studies of lung cancer, minimal labelling has been employed, and this approach has been recommended for serum proteomics [19]. However, saturation labelling has been successfully used as well [20–22].

The objective of this study was to compare protein abundance in blood plasma samples obtained from cancer patients before chemotherapy and after the second cycle of chemotherapy, using the 2D-DIGE approach. The control group was selected from patients with non-cancerous lung diseases. Moreover, we sought to determine whether particular lung cancers (adenocarcinoma [ADC] or SCC) are related to differences in blood plasma protein abundance.

## Materials and methods

### Sample collection

Informed consent was obtained from eight control patients (four men and four women, age range 52–76 years, mean age 63.2 ± 8.4 years) and eight non-small cell lung cancer (NSCLC) patients (four diagnosed with SCC and four diagnosed with ADC) before and after a second cycle of chemotherapy (five men and three women, age range 55–81, mean age 66 ± 8.2 years). Blood was collected from NSCLC patients one week before the first cycle of chemotherapy and one week before the third cycle of chemotherapy. The obtained venous blood samples were collected into tubes containing EDTA, and plasma was separated by centrifugation at 1,300 × g for 20 min at room temperature. Next, the plasma was transferred to sterile test tubes and re-centrifuged at 3,000 × g for 15 min at room temperature to remove residual cellular components. The plasma was transferred to cryotubes and stored at –80°C until the day of analysis.

Table 1 shows the clinical characteristics of the samples included in this work. The study was approved by the Ethics Committee of the Medical University of Bialystok (No. R-I-003/262/2004), and informed written consent for specimen collection was obtained from each patient before chemotherapy.

**Table 1:** Clinical and pathological characteristics of the patients whose samples were included in this study.

Sample		Age (y)	Gender	Diagnosis	Status/Response to chemotherapy	Stage	Smoking habit
Control 1		65	F	Pneumonia	Control		Active
Control 2		60	F	COPD	Control		Ex-smoker
Control 3		76	M	Pneumonia/asthma	Control		Active
Control 4		68	M	COPD	Control		Active
Control 5		71	M	bronchitis	Control		Ex-smoker
Control 6		53	F	asthma/bronchitis	Control		No
Control 7		61	F	COPD/asthma	Control		Ex-smoker
Control 8		52	M	bronchitis	Control		Active
Cancer 1B	Cancer 1A	57	M	SCC	Chemotherapy/PR	IA	Active
Cancer 2B	Cancer 2A	55	F	ADC	Chemotherapy/SD	IIIA	Active
Cancer 3B	Cancer 3A	65	F	ADC	Chemotherapy/PD	IIIA	Active
Cancer 4B	Cancer 4A	66	M	SCC	Chemotherapy/PR	IIIA	Active
Cancer 5B	Cancer 5A	66	M	ADC	Chemotherapy/SD	IIIB	Active
Cancer 6B	Cancer 6A	81	M	SCC	Chemotherapy/SD	IIIA	Active
Cancer 7B	Cancer 7A	73	M	ADC	Chemotherapy/SD	IIIA	Active
Cancer 8B	Cancer 8A	65	F	SCC	Chemotherapy/PR	IIIA	Active

COPD—Chronic Obstructive Pulmonary Disease, Clinical responses of patients after chemotherapy: PR—partial response, SD- stabilized disease, PD—progression of disease. Control 1–8 –blood plasma from control patients, Cancer 1-8B –blood plasma from lung cancer patients before first cycle of chemotherapy, Cancer 1-8A –blood plasma from lung cancer patients after second cycle of chemotherapy. Description of samples is the same as in Table 2.

### Immunodepletion of albumin and IgG from plasma

Blood plasma was depleted using albumin (Alb) and IgG depletion spin traps (GE Healthcare, Uppsala, Sweden). Blood plasma (50 μL) was combined with 50 μL binding buffer (0.15 M NaCl buffered with 20 mM phosphate buffer, pH 7.4). The samples were then applied to spin columns equilibrated with binding buffer. After a 5-min incubation period to allow the binding of Alb and IgG, the unbound protein fraction (depleted blood plasma) was washed with 270 μL of binding buffer. Proteins of the depleted blood plasma were precipitated using a 2-D Clean-up Kit (GE Healthcare). The precipitate was dissolved in 30 mM Tris, 7 M urea, 2 M thiourea, and 4% CHAPS. Protein concentrations were measured using the Coomassie Plus Kit (Thermo Scientific, Waltham, MA, USA) to evaluate the efficacy of depletion.

### Two-dimensional electrophoresis

A 2DE approach was used to test the efficacy of depletion. Samples of blood plasma and plasma after Alb and IgG depletion containing 500 μg of protein were resuspended in rehydration buffer (7 M urea, 2 M thiourea, 2% CHAPS, 2% immobilised pH gradient buffer, 40 mM dithiothreitol, and 0.002% bromophenol blue) to a final volume of 450 μL. Each sample was then loaded onto 24-cm Immobiline DryStrips with a 3 to 10 nonlinear pH range (GE Healthcare), and rehydrated for 10 h. Proteins were then separated by isoelectric focusing on an Ettan IPGphor apparatus (GE Healthcare) operating at 20°C with a current limited to 50 μA per strip and the following voltage program: 500 V/5 h; 1,000 V/1 h; 8,000 V/3 h; and 8,000 V/5.5 h. After isoelectric focusing, the strips were equilibrated for 15 min in SDS equilibration buffer (6 M urea, 75 mM Tris-HCl, pH 8.8, 29.3% glycerol, 2% SDS, and a trace of bromophenol blue) containing 10 mg/mL dithiothreitol, and then for 15 min in SDS equilibration buffer containing 25 mg/mL iodoacetamide. The equilibrated strips were then transferred to 12.5% polyacrylamide gels (25.5 × 19.6 cm, 1 mm thick) and sealed with 0.5% agarose. Second-dimension electrophoresis was then performed at 1 W/gel in an Ettan Dalt-Six apparatus (GE Healthcare) for 16 h. The gels were stained using Coomassie Brilliant Blue G250 (CBB-G250).

### Fluorescence labelling of samples with CyDyes and two-dimensional difference electrophoresis

Samples (50 μg) were dissolved in labelling buffer (7 M urea, 2 M thiourea, 4% [wt/vol] CHAPS, and 30 mM Tris) and labelled with CyDye DIGE Fluor minimal dyes (GE Healthcare) reconstituted in fresh 99.8% anhydrous dimethylformamide at a concentration of 50 μg protein to 400 pmol fluor dye [23]. The labelling reaction was performed in the dark on ice for 30 min. Experimental samples of blood plasma from the control patients and lung cancer patients before and after a second cycle of chemotherapy were labelled with Cy3 and Cy5 according to the scheme presented in Table 2.

**Table 2:** Mixing and dying scheme of blood plasma samples of control patients and lung cancer patients before and after a second cycle of chemotherapy; n = 8 for each group.

Gel no	Cy2	Cy3	Cy5
**1**	Pooled Std.	Control 1	Cancer 5A (ADC)
**2**	Pooled Std.	Cancer 1B (SCC)	Control 5
**3**	Pooled Std.	Cancer 1A (SCC)	Cancer 5B (ADC)
**4**	Pooled Std.	Control 2	Cancer 6A (SCC)
**5**	Pooled Std.	Cancer 2B (ADC)	Control 6
**6**	Pooled Std.	Cancer 2A (ADC)	Cancer 6B (SCC)
**7**	Pooled Std.	Control 3	Cancer 7A (ADC)
**8**	Pooled Std.	Cancer 3B (ADC)	Control 7
**9**	Pooled Std.	Cancer 3A (ADC)	Cancer 7B (ADC)
**10**	Pooled Std.	Control 4	Cancer 8A (SCC)
**11**	Pooled Std.	Cancer 4B (SCC)	Control 8
**12**	Pooled Std.	Cancer 4A (SCC)	Cancer 8B (SCC)

Control 1–8 –blood plasma from control patients. Cancer 1-8B –blood plasma from lung cancer patients (consisting of four SCC and four ADC) before first cycle of chemotherapy. Cancer 1-8A –blood plasma from lung cancer patients (consisting of four SCC and four ADC) after second cycle of chemotherapy.

For 2D DIGE protocols [24], the calculated minimum number of gels to be run for our 2D DIGE experiment for 8 patients is 12 gels. This was calculated according to the formula:

$$No.\;of\;gels=\frac{\text{no}.\;\text{of}\;\text{groups}\;\times \;\text{no}.\;\text{of}\;\text{biological}\;\text{reps}\;}{2\;\left(since\;two\;groups\;on\;each\;gels\right)}$$

For our experiment, the number of groups was 3 (control, before chemotherapy, and after chemotherapy), and the number of biological replicates (patient samples) was 8, so number of gels was (3 x 8)/2 = 12.

Cy2 dye was used to label a pooled sample comprising equal amounts of each of the samples within the experiment, and acts as an internal standard. An equal amount of Cy2-labelled pooled standard was loaded on each gel for normalisation and to correct for gel-to-gel variability. After the labelling reaction, differentially labelled samples (50 μg of Cy2, Cy3, and Cy5-labelled samples) were mixed together according to the scheme presented in Table 2. Rehydration buffer was then added to each sample mixture to reach a final volume of 450 μL. Differentially labelled samples were then loaded on 24-cm Immobiline DryStrips, with a 3 to 10 nonlinear gradient pH range (GE Healthcare) and rehydrated for 12 h. Proteins were then separated by isoelectric focusing and SDS-PAGE, as described above.

### Image acquisition and analysis

After electrophoresis, the gels were scanned with a Typhoon 9500 FLA scanner (GE Healthcare) using the parameters suggested by the manufacturer for 2D-DIGE experiments. The scanned images were analysed with DeCyder Differential In-Gel Analysis version 5.02 software (GE Healthcare) to identify the fluorescence intensities of the spots. The DeCyder biological variation analysis module was used to detect protein spots, simultaneously matching all 24 protein spot maps from 12 gels using the following parameters: the estimated number of spots was set to 10,000 and the minimum spot size was set to 3,000. Protein spots with a p-value <0.05 by one-way ANOVA analysis, which showed an increase or decrease in relative intensity, were considered to be differentially abundant proteins. Only spots that were successfully matched on >80% of the gel images were considered. To properly select and identify the spots, gels were stained using CBB-G250 after 2D-DIGE, followed by spot excision and identification using matrix-assisted laser desorption/ionisation time-of-flight/time-of-flight (MALDI-TOF/TOF) mass spectrometry (MS).

### Protein identification by mass spectrometry

Protein spots indicated by statistical analysis were excised from the gels, put in Eppendorf tubes, and washed with 50 μL of 50 mM ammonium bicarbonate. The wash was discarded and the spots were washed again with 50 μL of 50 mM ammonium bicarbonate in 50% acetonitrile solution, and incubated for 5 min in room temperature. After discarding the wash and drying the spots, 2 μL of 0.2 μg/μL modified sequencing grade trypsin (Promega, Madison, WI, USA) solution and 2 μL of 50 mM ammonium bicarbonate were added and the samples incubated for 12 h at 37°C. After digestion, the spots were placed in 100 μL of 0.1 trifluoroacetic acid (TFA) and desalted with Zip-Tip C-18 pipette tips (Millipore, Billerica, MA, USA; [25]). Each Zip-Tip was first washed with 100% acetonitrile and then equilibrated with 50% acetonitrile in 0.1% TFA and 0.1% TFA in water. After washing and equilibration, the peptides were loaded onto the Zip-Tip and then eluted with 2 μL of 50% acetonitrile in 0.1% TFA. The eluted samples were mixed with 2 μL of the matrix solution (5 mg α-cyano-4-hydroxycinnamic acid [Bruker Daltonics, Bremen, Germany] in 1 mL of 50% acetonitrile in 0.1% TFA), and half of this mixture was spotted onto the matrix-assisted laser desorption/ionisation target plate (MT 34 Target Plate Ground Steel; Bruker Daltonics) and left to dry. MALDI-TOF/TOF MS analysis was performed using a MALDI-TOF tandem mass spectrometer (Autoflex Speed; Bruker Daltonics). Collected MS and tandem MS LIFT spectra of selected ions were externally calibrated using monoisotopic protonated ion peptide calibration standards (Bruker Daltonics), and imported to BioTools (Bruker Daltonics). The MS peptide mass fingerprint (PMF) and fragment mass spectra (MS/MS) from each individual spot were combined and used to search against the National Centre for Biotechnology Information Homo sapiens database (searched on December 4, 2017) using the Mascot Server (Matrix Science, London, UK) with the following settings: cleavage enzyme, trypsin; max missed cleavages, 2; fragment ion mass tolerance, 0.5 Da; parent ion mass tolerance, 200 ppm; alkylation of cysteine by carbamidomethylation as a fixed modification; and oxidation of methionine as a variable modification.

### Validation of 2D–DIGE results by Western blot

Western-blot technique was used to validate the results obtained in proteomics study. We used V3 stain-free workflow which eliminates the need for stripping and reprobing the blot for housekeeping proteins [26]. The expression of three proteins of interest was evaluated in serum (i) of control patients and lung cancer patients before and after second cycle of chemotherapy (transferrin, fibrinogen α chain), as well as (ii) lung cancer patients before chemotherapy in relation to SCC and ADC (vitronectin). The Western blot was performed as described by Repetto *et al*. [27] with some modifications. Equal amounts of protein (10 μg) were fractionated on 12% Criterion™ TGX Stain-Free™ Protein Gels (Bio-Rad, Hercules, CA, USA). After electrophoresis, gels were activated on a Chemidoc according to manufacturer instructions (Bio-Rad), then transferred to PVDF membranes using Mini Trans—Biol Cell (Bio-Rad) at 60 V for 90 min. After transfer, a stain-free image of PVDF membranes for total protein normalization was obtained before membranes were rinsed briefly in distilled water and blocked with 5% bovine serum albumin (Sigma-Aldrich, St. Louis, MO, USA), then incubated with primary polyclonal antibodies (Abcam, Cambridge, UK) against transferrin (1:10000), fibrinogen (1:5000), vitronectin (1:1000) overnight at 4°C. After rinsing the membrane to remove unbound primary antibodies, it was exposed to goat anti-rabbit antibodies (1:5000; Sigma) linked to alkaline phosphatase. Products were visualized by incubation in a solution of alkaline phosphate buffer with an addition of NBT (Sigma) and BCIP (Sigma) in the dark. Antibody-bound proteins were detected by enhanced chemiluminescence using the Chemidoc Imaging System (Bio-Rad). All band intensities were measured with Image Lab Software Version 5.2 (Bio-Rad, Hercules). The image of the gel acquired before its transfer was used as control for equal protein loading among samples. The volume density of each target protein band was normalized to its respective total protein content, whereas total protein band was normalized to the total protein loaded into each lane using stain-free technology with data expressed in arbitrary units.

### Functional analysis

Ingenuity pathway analysis (IPA; IngenuityR Pathway Analysis, IPAR, Qiagen, Redwood City, CA, USA) software was used to investigate the functional and canonical pathways that were enriched in the differentially expressed proteins (http://www.ingenuity.com). Fisher’s exact test and Benjamini–Hochberg multiple testing corrections were used to calculate statistical significance (p < 0.05).

### Statistical analysis

Statistical analysis of changes in protein abundance was performed using the Biological Variance Module of DeCyder Differential In Gel Analysis version 5.02 software (GE Healthcare) on eight biological replicates (individual patients). Direct comparisons of spot volumes were made between the Cy3- or Cy5-labelled samples and the Cy2-labelled pool standard for each gel. The Cy3/Cy2 and Cy5/Cy2 ratio was used to calculate average changes in abundance. Data are expressed as log standardised abundances to ensure a normal distribution of the data. One-way ANOVA, t-test and the average ratio test were performed; changes in protein spot abundance were considered statistically significant at p < 0.05. For the MS PMF and MS/MS ion search, statistically significant (p ≤ 0.05) matches by MASCOT were regarded as correct hits.

## Results

### Depletion of albumin and IgG from blood plasma

**Fig 1:**
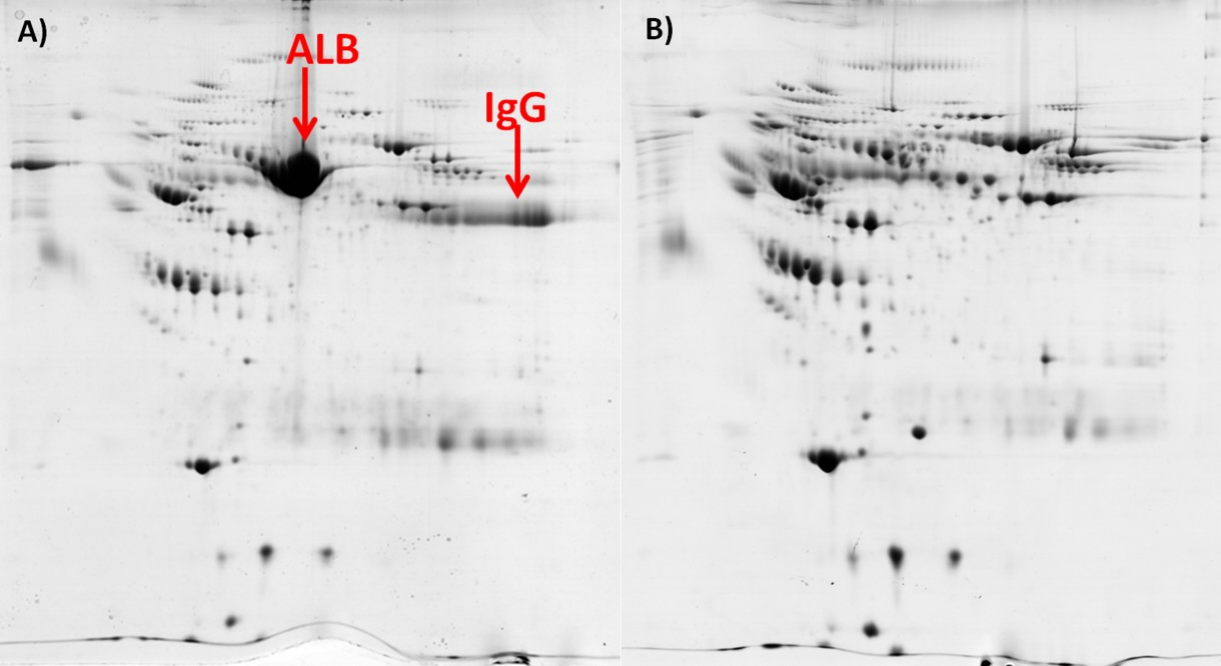
Electropherograms of blood plasma proteins. A–before depletion, B–after depletion of albumin (ALB) and IgG, with the use of spin trap columns.

Depletion of Alb and IgG from blood plasma decreased the amount of protein by 73%, from 3.02 ± 0.27 mg applied to the spin trap column to 0.83 ± 0.09 mg recovered after depletion (n = 24). Electropherograms indicated that the Alb and IgG fractions were not visible by CBB staining in the depleted samples (Fig 1).

### 2D-DIGE analysis of differentially expressed proteins in the blood plasma of control patients and lung cancer patients before chemotherapy

#### Comparison of all patients

**Fig 2:**
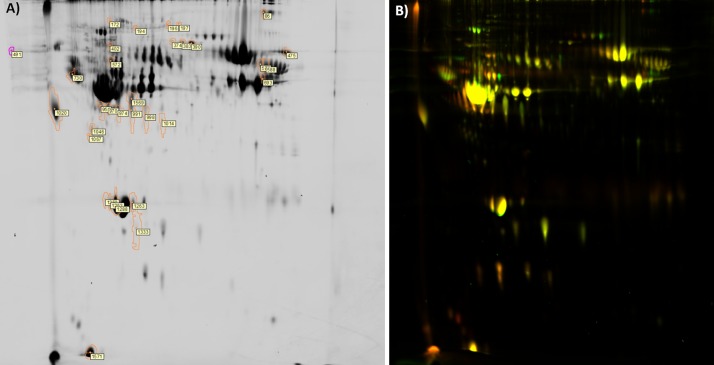
Representative 2D-DIGE profiling of blood plasma from control patients vs. lung cancer patients, before chemotherapy. A–protein staining, B–overlay of control and cancer samples. Thirty-two spots (numbers correlate with descriptions in Table 3) with significantly different abundance between control patients and cancer patients before chemotherapy are shown (p < 0.05). Eight spots could not be identified.

Out of 32 differentially abundant spots, we identified 24 differentially expressed proteins or proteoforms (eight spots could not be identified) in the blood plasma of control patients compared with all NSCLC patients before chemotherapy (Table 3 and Fig 2). Control plasma was characterised by a higher abundance of complement C3, coagulation factor XII, fibrinogen β chain, prothrombin isoform 2, gelsolin isoform e, proapolipoprotein, inter-α-globulin inhibitor H4, α-2-HS-glycoprotein, α-2-macroglobulin isoforms a and b, and protein SP40,40. On the other hand, the plasma of lung cancer patients before chemotherapy was characterised by a higher abundance of fibrinogen α chain, zinc-α-glycoprotein precursor, five proteoforms of haptoglobin, and orsomucoid 1. Although the samples included both cancer stages I and III proteomic changes were similar regardless of stage. We have provided examples with magnified regions of all gels for samples at stage I and stage III lung cancer with differential protein expression, as well as line charts of the selected spots (charts generated from DeCyder software) in S1 Table. These data confirm similar changes in the blood proteome regardless of cancer stage.

**Table 3:** Proteins found to be present in different abundances in the depleted serum of control patients and lung cancer patients before chemotherapy and after second cycle of chemotherapy.

Spot no	Protein	Gene name	Accession no.	Mass /pI (theoretical)	Protein score	Sequence coverage (%)	Number of peptides (ion score ≥30)	Before chemotherapy vs Control^a^	After chemotherapy vs Control^b^	After chemotherapy vs Before^c^
86	complement C3 preproprotein [*Homo sapiens*]	C3	NP_000055.2	188569/6.02	574	22	6	-2.26		
172	inter-alpha (globulin) inhibitor H4 (plasma Kallikrein-sensitive) [*Homo sapiens*]	ITIH4	EAW65265.1	101521/6.21	135	15	2	-1.20		
196	alpha-2-macroglobulin isoform a precursor *[Homo sapiens*]	A2M	NP_000005.2	164614/6.00	432	15	5	-1.26		
197	alpha-2-macroglobulin isoform b [*Homo sapiens*]	A2M	NP_001334353.1	153716/6.08	316	15	4	-1.22		
402	prothrombin isoform 2 [*Homo sapiens*]	F2	NP_001298186.1	69757/5.55	360	35	3	-1.24		
693	fibrinogen beta chain, isoform CRA_d [*Homo sapiens*]	FGB	EAX04933.1	52759/8.33	241	46	2	-1.63		
1014	haptoglobin, partial [*Homo sapiens*]	HP	AAC27432.1	38722/6.14	325	26	4	1.53		
1046	SP40,40, partial [*Homo sapiens*]	CLU	AAA60567.1	36997/5.74	319	31	3	-1.30		
200	alpha-2-macroglobulin isoform b [*Homo sapiens*]	A2M	NP_001334353.1	153716/6.08	472	19	5		1.22	
272	inter-alpha-trypsin inhibitor heavy chain H4 isoform 2 precursor [*Homo sapiens*]	ITIH4	NP_001159921.1	100023/6.06	249	21	3		-1.35	
570	hemopexin precursor [*Homo sapiens*]	HPX	NP_000604.1	52385/6.55	562	48	6		1.19	
525	fibrinogen alpha chain preproprotein, isoform alpha [*Homo sapiens*]	FGA	AAK31372.1	70666/8.38	460	39	4		-1.30	
843	immunoglobulin heavy chain constant alpha 1 membrane bound isoform 1, partial [*Homo sapiens*]	IGHA1	ACA51870.1	19738/4.94	146	37	2		1.15	
923	leucine-rich alpha-2-glycoprotein precursor [*Homo sapiens*]	LRG1	NP_443204.1	38382/6.45	527	34	6		1.44	
1032	alpha-2-macroglobulin isoform b [*Homo sapiens*]	A2M	NP_001334353.1	153716/6.08	316	15	4		-1.45	
1066	Clusterin, partial [*Homo sapiens*]	CLU	AAN78322.1	16267/5.60	117	2626	2		-1.42	
1091							2		-1.43	
1068	SP40,40, partial [*Homo sapiens*]	CLU	AAA60567.1	36997/5.74	430	32	4		-1.45	
1019	complement C3 preproprotein [*Homo sapiens*]	C3	NP_000055.2	188569/6.02	236	9	4			-1.41
374	coagulation factor XIII, B polypeptide [*Homo sapiens*]	F13B	AAT85802.1	77723/5.97	308	27	5	-1.38	-1.40	
383	gelsolin isoform e [*Homo sapiens*]	GSN	NP_001244959.1	81776/5.58	288	36	2	-1.35	-1.46	
390					325	38	1	-1.40		
571	fibrinogen alpha chain preproprotein, isoform alpha [*Homo sapiens*]	FGA	AAK31372.1	70666/8.38	357	41	1	1.58	1.61	
588	fibrinogen alpha chain isoform alpha precursor [*Homo sapiens*]	FGA	NP_068657.1	70227/8.23	562	29	6	1.48	1.24	
602					411	29	5		1.31	
618					447	35	5		1.31	
730	alpha-2-HS-glycoprotein, isoform CRA_a [*Homo sapiens*]	AHSG	EAW78187.1	39970/5.43	198	22	2	-1.21	-1.21	
954	zinc-alpha2-glycoprotein precursor [*Homo sapiens*]	AZGP1	BAA14417.1	34079/5.57	425	47	5	1.52	1.68	
970	apolipoprotein A-IV precursor, partial [*Homo sapiens*]	APOA4	AAA51748.1	43358/5.22	473	40	4	1.50	1.70	
974					266	28	3	1.55	1.70	
870	HP protein [*Homo sapiens*]	HP	AAH70299.1	31647/8.48	351	32	4		1.34	
880					285	37	3		1.45	
991					351	32	4	1.50	1.56	
998					282	35	3	1.69		
1569					229	28	2	1.47	1.49	
1020	Orosomucoid 1 [*Homo sapiens*]	ORM1	AAI43315.1	23817/4.93	199	24	3	1.43	1.43	
1046	SP40,40, partial [*Homo sapiens*]	CLU	AAA60567.1	36997/5.74	319	31	3	-1.30		
1068					430	32	4		-1.45	
1263	proapolipoprotein, partial [*Homo sapiens*]	APOA1	AAA51747.1	28944/5.45	519	59	4	-1.24	-1.36	
1252					159	41	1	-1.37	-1.43	
1570					647	60	6		-1.31	
1333	haptoglobin, partial [*Homo sapiens*]	HP	ALX40934.1	13744/6.10	267	68	2	3.00	3.29	
486	transferrin variant, partial [*Homo sapiens*]	TF	BAD96475.1	79310/6.68	603	35	7		-1.31	
491					666	39	7		-1.37	-1.20
493					231	26	2		-1.38	-1.32
496					461	43	4		-1.29	
534	PREDICTED: serotransferrin isoform X1 [*Homo sapiens*]	TF	XP_016862578.1	79294/6.81	331	31	4		-1.29	
540					433	33	6		-1.61	-1.46
1572					483	35	5		-1.38	-1.30
515	hemopexin precursor, partial [*Homo sapiens*]	HPX	AAA52704.1	52254/6.57	515	37	4		1.25	
551					349	41	3		1.24	
585					562	50	5		1.15	
887					260	39	2		1.20	1.12
1470	hemoglobin beta chain variant Hb S-Wake [*Homo sapiens*]	HBB	AAN11320.1	16045/7.12	575	73	5		-2.37	-1.41

^a^ A negative average ratio denotes a higher abundance of proteins in control patients, whereas a positive average ratio denotes a higher abundance of proteins in lung cancer patients before chemotherapy.

^b^ A negative average ratio denotes a higher abundance of proteins in control patients, whereas a positive average ratio denotes a higher abundance of proteins in cancer patients after second cycle of chemotherapy.

^c^ A negative average ratio denotes a higher abundance of proteins in patients before chemotherapy, whereas a positive average ratio denotes a higher abundance of proteins in cancer patients after second cycle of chemotherapy.

#### Comparative analysis of ADC and SCC patients

We re-analysed our results taking the specific lung cancer diagnosis into consideration. Eight spots discriminating the proteome of SCC patients from that of ADC patients were identified (Table 4). The plasma of SCC patients was characterised by a higher abundance of vitronectin, coagulation factor XIII, plasminogen, and gelsolin. On the other hand, the plasma of ADC patients was characterised by a higher abundance of transferrin, immunoglobin heavy chain constant region mu, and leucine-rich alpha-2-glycoprotein precursor.

**Table 4:** Proteins found to be present in different abundances in the depleted serum of lung cancer patients before chemotherapy and after second cycle of chemotherapy, in relation to SCC and ADC.

Spot no	Protein	Gene name	Accession no.	Mass /pI (theoretical)	Protein score	Sequence coverage (%)	Number of peptides (ion score ≥30)	Before chemotherapy SCC /ADC^a^	After chemotherapy SCC /ADC^b^
267	Plasminogen [*Homo sapiens*]	PLG	AAH60513.1	93263/6.89	770	45	10	1.39	
549	Vitronectin [*Homo sapiens*]	VTN	AAH05046.1	55099/5.55	403	32	4	1.80	
553					368	32	5	1.77	
897	immunoglobulin heavy chain constant region mu, partial [*Homo sapiens*]	IGHM	CAC20458.1	50117/6.40	327	24	5	-2.06	
923	leucine-rich alpha-2-glycoprotein precursor [*Homo sapiens*]	LRG1	NP_443204.1	38382/6.45	527	34	6	-1.44	
1343	Haptoglobin hp2-alpha, partial [*Homo sapiens*]	HP	CAA25248	42126/6.25	180	24	2		-1.54
374	coagulation factor XIII, B polypeptide [*Homo sapiens*]	F13B	AAT85802.1	77723/5.97	308	27	5	1.19	1.25
383	gelsolin isoform e [*Homo sapiens*]	GSN	NP_001244959.1	81776/5.58	288	36	2	1.22	1.26
493	transferrin variant, partial [*Homo sapiens*]	TF	BAD96475.1	79310/6.68	231	26	2	-1.50	-1.49

^a^ A negative average ratio denotes a higher abundance of proteins in ADC patients, whereas a positive average ratio denotes a higher abundance of proteins in SCC patients before chemotherapy.

^b^ A negative average ratio denotes a higher abundance of proteins in ADC patients, whereas a positive average ratio denotes a higher abundance of proteins in SCC cancer patients after second cycle of chemotherapy.

### 2-DIGE analysis of differentially expressed proteins in the blood plasma of control patients and lung cancer patients after second cycle of chemotherapy

#### Comparison of all patients

We identified 41 differentially expressed proteins or proteoforms in the blood plasma of control patients compared with NSCLC patients after the second cycle of chemotherapy (Table 3). However, 13 proteins or proteoforms were the same as those detected before chemotherapy. The control plasma contained higher abundances of proapolipoprotein, coagulation factor XIII, clusterin, fibrinogen α chain, hemoglobin beta chain, inter-α-trypsin inhibitor, α-2-macroglobulin isoform b, protein SP40,40, four proteoforms of transferrin, and three proteoforms of serotransferrin X1. On the other hand, the plasma of lung cancer patients after chemotherapy was characterised by a higher abundance of two forms of apolipoprotein A-IV precursor, four forms of fibrinogen α chain, five forms of hemopexin, haptoglobin, α-2-macroglobulin isoform b, immunoglobulin heavy chain constant α 1 membrane bound isoform 1, and leucine-rich α-2 glycoprotein precursor.

#### Comparative analysis of ADC and SCC patients

We identified four spots discriminating the proteome of SCC patients from that of ADC patients (Table 4). Three proteins discriminating SCC from ADC after a second cycle of chemotherapy were confirmed, including coagulation factor XIII and gelsolin. Transferrin and haptoglobin hp2 abundance was higher in the blood plasma of ADC patients than in that of SCC patients.

### 2-DIGE analysis of proteins differentially expressed in the blood plasma of lung cancer patients before and after second cycle of chemotherapy

#### Comparison of all patients

The average numbers of protein spots (mean ± SD) and CV (%) in the control group, in lung cancer patients before chemotherapy, and in lung cancer patients after the second cycle of chemotherapy were 1375 ± 136 (9.9%), 1341 ± 156 (11.6%), and 1270 ± 100 (7.9%), respectively. We identified seven differentially expressed proteins or proteoforms in the blood plasma of NSCLC patients before chemotherapy compared with after the second cycle of chemotherapy (Table 3). Plasma before chemotherapy was characterised by a higher abundance of complement C3 preproprotein, hemoglobin β chain variant S-Wake, two variants of transferrin, and two variants of serotransferrins isoform X1. After a second cycle of chemotherapy, blood plasma contained a higher abundance of hemopexin precursor.

#### Comparative analysis of ADC and SCC patients

Two proteins were differently expressed in ADC patients before chemotherapy and after the second cycle of chemotherapy; hemopexin was more abundant before chemotherapy and fibrinogen gamma was more abundant after chemotherapy (Table 5). For SCC patients, five detected spots were more abundant after chemotherapy, including the haemoglobin beta chain, complement C3, transferrin, and two variants of serotransferrins.

**Table 5:** Proteins found to be present in different abundances in the depleted serum of lung cancer patients before and after second cycle of chemotherapy, in relation to ADC and SCC.

Spot no	Protein	Gene name	Accession no.	Mass /pI (theoretical)	Protein score	Sequence coverage (%)	Number of peptides (ion score ≥30)	Average ratio ADC before/after chemotherapy	Average ratio SCC before/after chemotherapy
887	hemopexin precursor, partial [*Homo sapiens*]	HPX	AAA52704.1	52254/6.57	260	39	2	-1.16	
611	fibrinogen gamma chain isoform gamma-B precursor [*Homo sapiens*]	FGG	NP_068656.2	52106/5.37	402	37	5	1.52	
1470	hemoglobin beta chain variant Hb S-Wake [*Homo sapiens*]	HBB	AAN11320.1	16045/7.12	575	73	5		4.11
1019	complement C3 preproprotein [*Homo sapiens*]	C3	NP_000055.2	188569/6.02	303	10	4		1.57
1572	PREDICTED: serotransferrin isoform X1 [*Homo sapiens*]	TF	XP_016862578.1	79294/6.81	433	33	6		1.31
467	transferrin variant, partial [*Homo sapiens*]	TF	BAD96475.1	79310/6.68	603	35	7		1.32
534	PREDICTED: serotransferrin isoform X1 [*Homo sapiens*]	TF6	XP_016862578.1	79294/6.81	331	31	4		1.22

A negative average ratio denotes a higher abundance of proteins in patients before chemotherapy, whereas a positive average ratio denotes a higher abundance of proteins in cancer patients after second cycle of chemotherapy.

**Fig 3:**
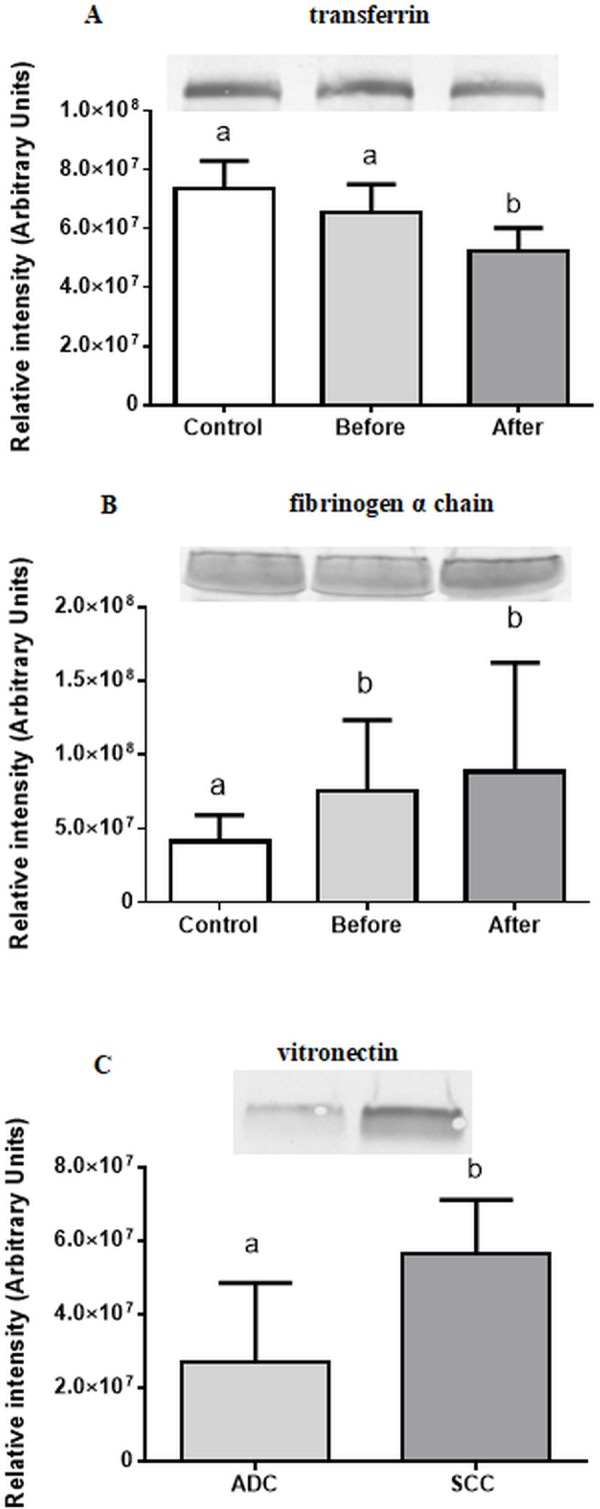
**Immunoblotting validation of transferrin (A), fibrinogen α chain (B) and vitronectin (C) of control and lung cancer serum samples before and after a second cycle of chemotherapy.** Results are expressed as means ± SD. For transferrin and fibrinogen representative blots for one patient are shown. For vitronectin blots for 4 ADC patients and 4 SCC patients are shown. Different superscripts indicate significant differences between the serum samples of control patients and cancer patients before and after chemotherapy (p < 0.05).

Using Western blot we confirmed further corroborate the decrease in transferrin in serum of lung cancer patients after a second cycle of chemotherapy (Fig 3A); an increase in content of fibrinogen α chain in lung patients before and after second cycle of chemotherapy (Fig 3B) and decrease in the content of vitronectin in serum of lung cancer patients before chemotherapy in relation to SCC and ADC (Fig 3C). The images of the gels after SDS-PAGE electrophoresis and membranes after transfer and before incubation with primary antibodies are shown in S1 Fig.

#### Changes in protein abundance across individual patients

S2 Table summarises the observed changes in expression of selected proteins (493, 887, 1019, 1572) across gels with blood plasma from lung cancer patients before the first cycle and after the second cycle of chemotherapy. Generally, proteins, including transferrin (spot no. 493) changed in the same way in samples before and after the second cycle of chemotherapy. However, outliers could be found for each of the presented spots originating from different samples, for example 55263 Cy5 and 55260 Cy5 for spot no. 493, 55269 Cy5 for spot no. 887, 55262 Cy3 for spot no. 1019, and 55262 Cy3 and 55263 Cy5 for spot no. 1572; this could reflect patient-specific protein expression patterns.

### Ingenuity pathway analysis

**Fig 4:**
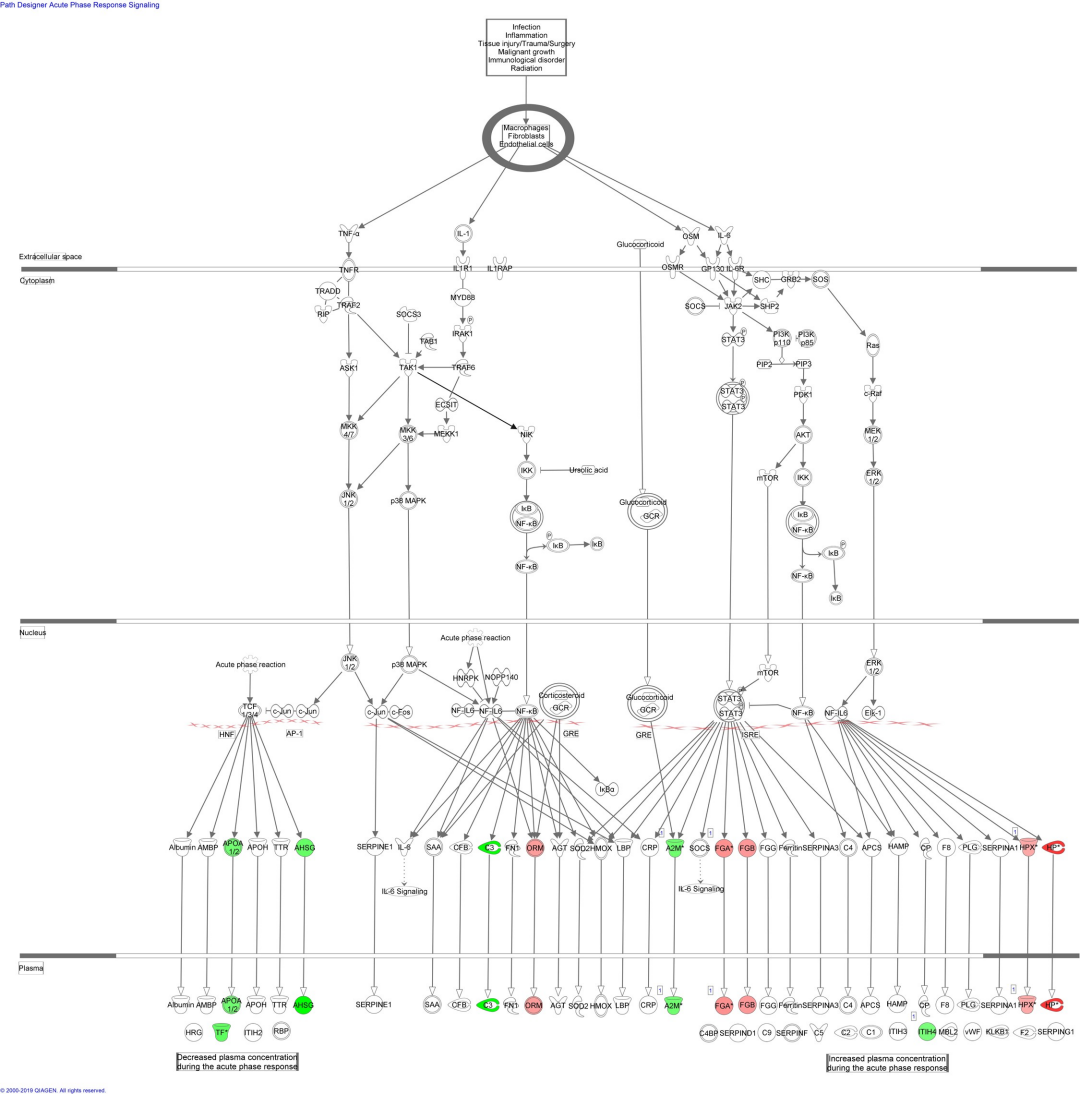
Acute phase response signalling pathway overlap with differentially expressed blood proteins between control patients and lung cancer patients before and after second cycle of chemotherapy. Red proteins are upregulated in cancer patients, green proteins are downregulated in cancer patients. White-proteins were not identified in our proteomics study, but are incorporated as part of the network.

**Fig 5:**
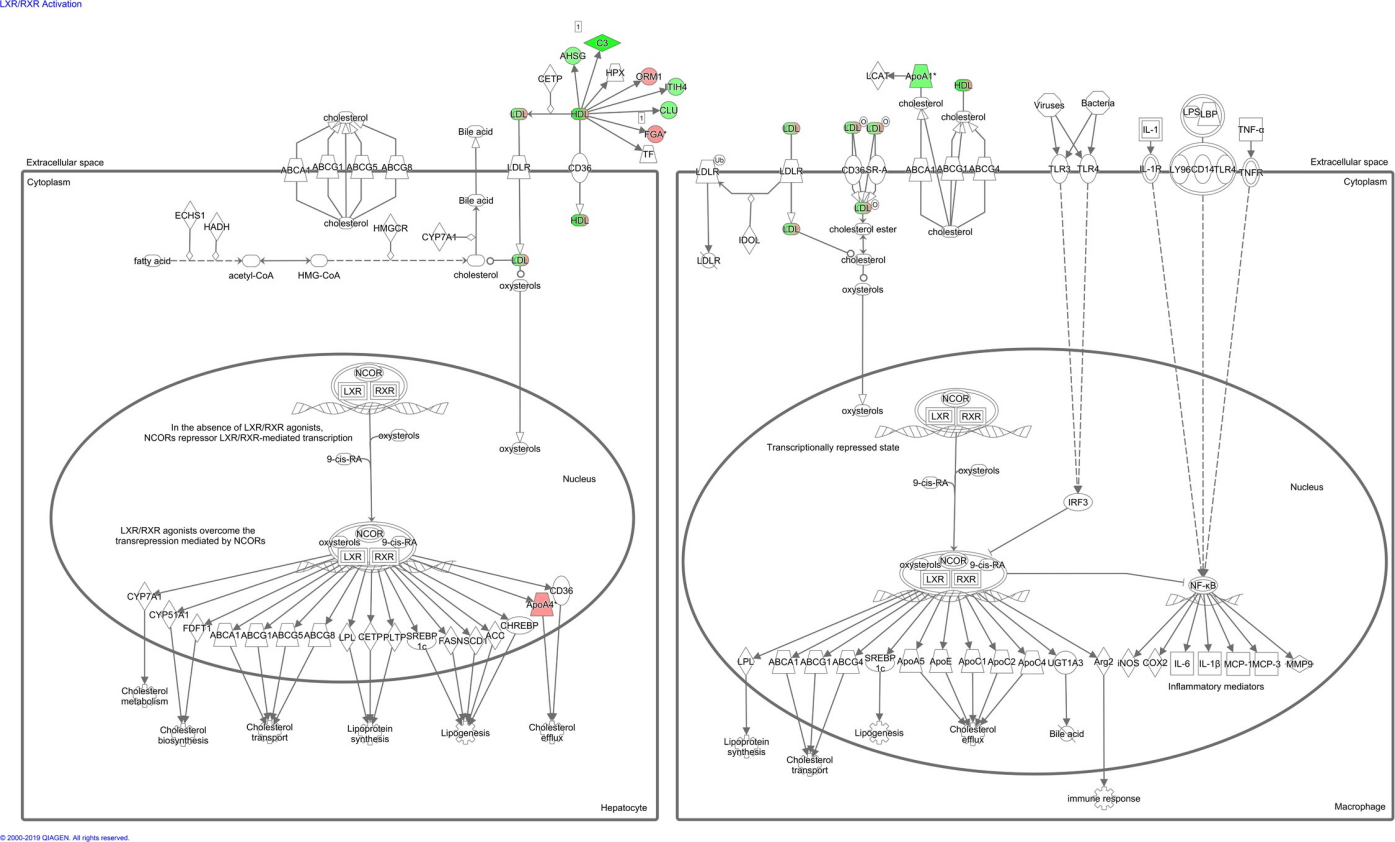
LXR/RXR activation pathway overlap with differentially expressed blood proteins between control patients and lung cancer patients before and after second cycle of chemotherapy. Red proteins are upregulated in cancer patients, green proteins are downregulated in cancer patients. White proteins were not identified in our proteomics study, but are incorporated as part of the network.

**Fig 6:**
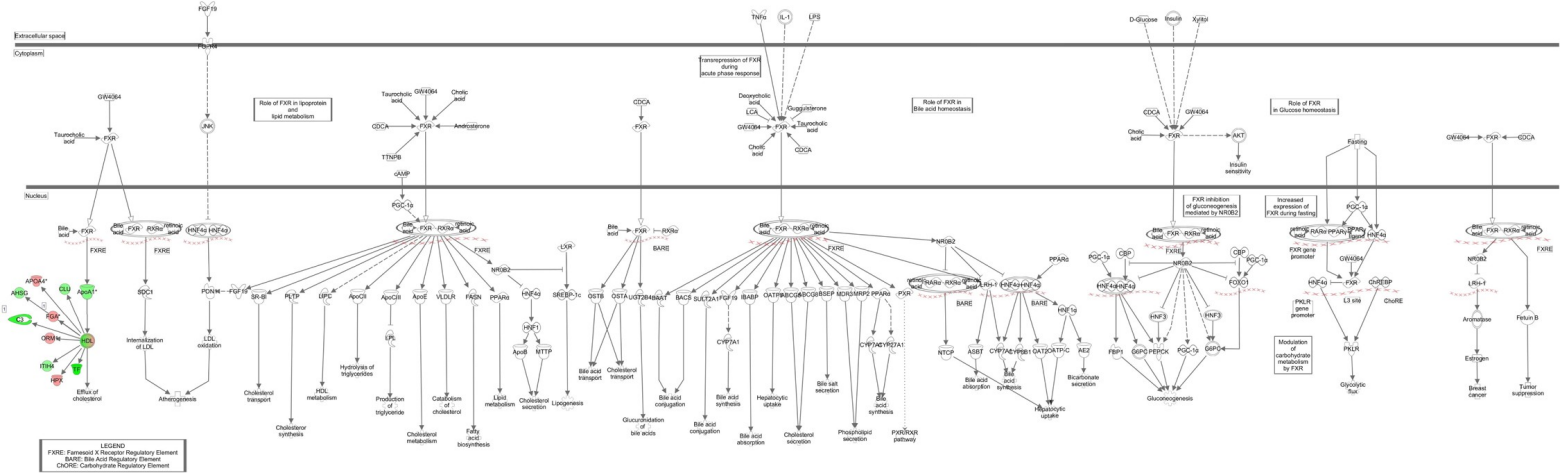
RXR/RXR activation pathway overlap with differentially expressed blood proteins between control patients and lung cancer patients before and after second cycle of chemotherapy. Red proteins are upregulated in cancer patients, green proteins are downregulated in cancer patients. White proteins were not identified in our proteomics study, but are incorporated as part of the network.

**Fig 7:**
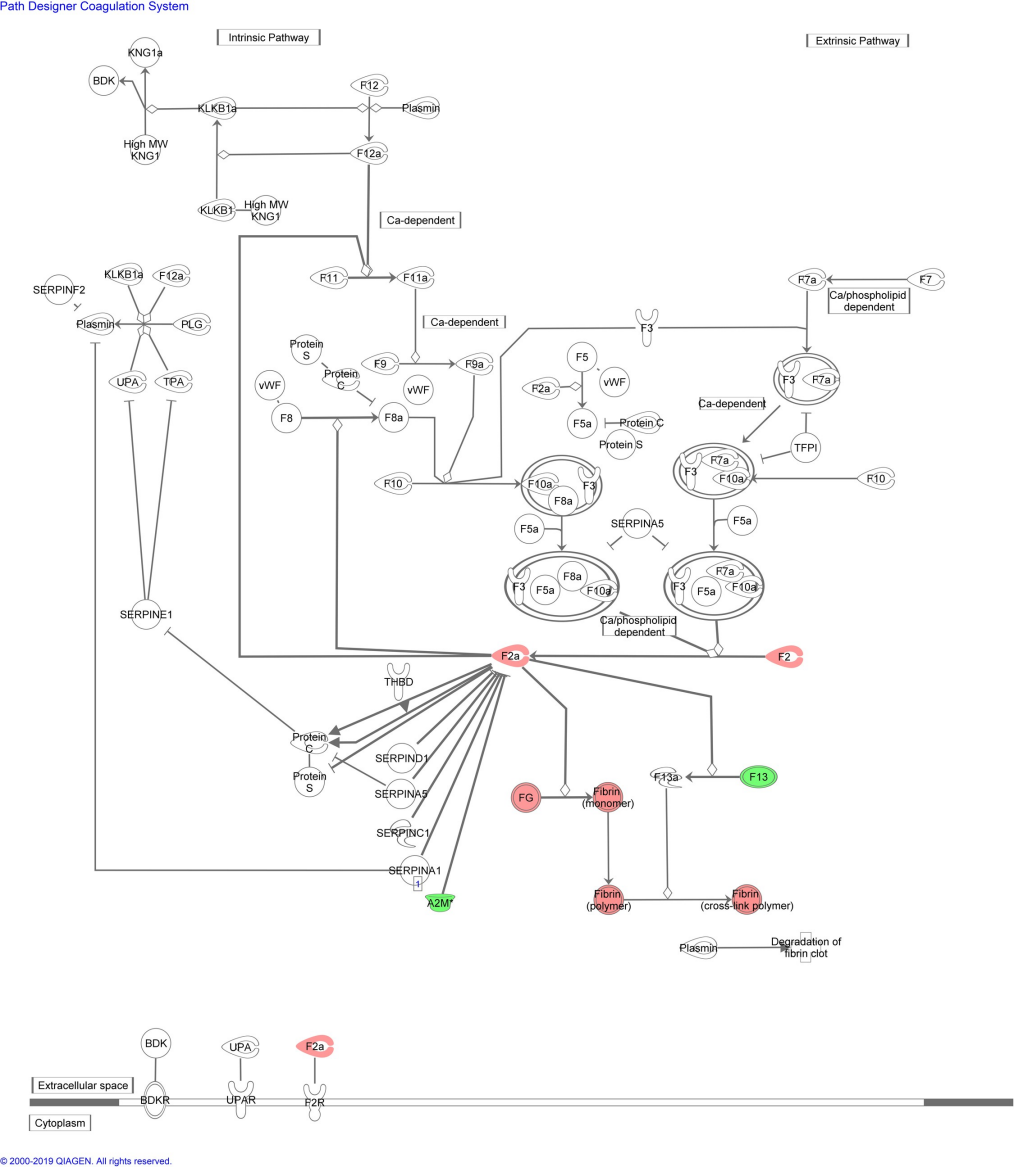
Coagulation system pathway overlap with differentially expressed blood proteins between control patients and lung cancer patients before and after second cycle of chemotherapy. Red proteins are upregulated in cancer patients, green proteins are downregulated in cancer patients. White proteins were not identified in our proteomics study, but are incorporated as part of the network.

A summary of the IPA for differentially expressed proteins in blood from control and lung cancer patients before chemotherapy is provided in Table 6. The most significant enriched canonical pathways included “acute phase response signaling”, “FXR/RXR and LXR/RXR activation”, and “coagulation system”. Figs 4–7 depict differentially expressed proteins mapped to the most significant enriched canonical pathways. The molecular and cellular function lists included “cell-to-cell signalling and interaction”, lipid metabolism”, “molecular transport”, and “free radical scavenging”. The physiological system development and function lists included “haematological system development and function” and “immune response”.

**Table 6:** Functional analysis (IPA) of differentially abundant proteins between the blood plasma of control patients and lung cancer patients before chemotherapy.

**Ingenuity Canonical Pathways**	**Molecules**
Acute Phase Response Signaling	HP, APOA1, C3, ORM1, ITIH4, AHSG, FGB, FGA, A2M, F2
LXR/RXR and FXR/RXR Activation	APOA1, C3, APOA4, ORM1, ITIH4, AHSG, FGA, CLU
Coagulation System	FGB, FGA, F13B, A2M, F2
**Molecular and cellular function**	**Molecules**
Cell-To-Cell Signaling and Interaction	A2M, APOA1, APOA4, C3, CLU, F2, FGA, ORM1, FGB,GSN, AHSG
Lipid Metabolism and Molecular Transport	APOA1, APOA4, CLU, F2, GSN, HP, C3, AZGP1, ORM1, AHSG, A2M, FGA, FGB, HP
Free Radical Scavenging	APOA1, APOA4, C3, F2, GSN, HP, ITIH4, CLU
**Physiological system development and Function**	**Molecules**
Hematological System Development and Function	A2M,APOA1,APOA4,C3,CLU,F2,FGA,ORM1, HP, FGB, GSN, F13B, AHSG
Immune Cell Trafficking and Tissue morphology	A2M,APOA1,APOA4,C3,CLU,F2,FGA,ORM1, FGB,GSN, AHSG

**Fig 8:**
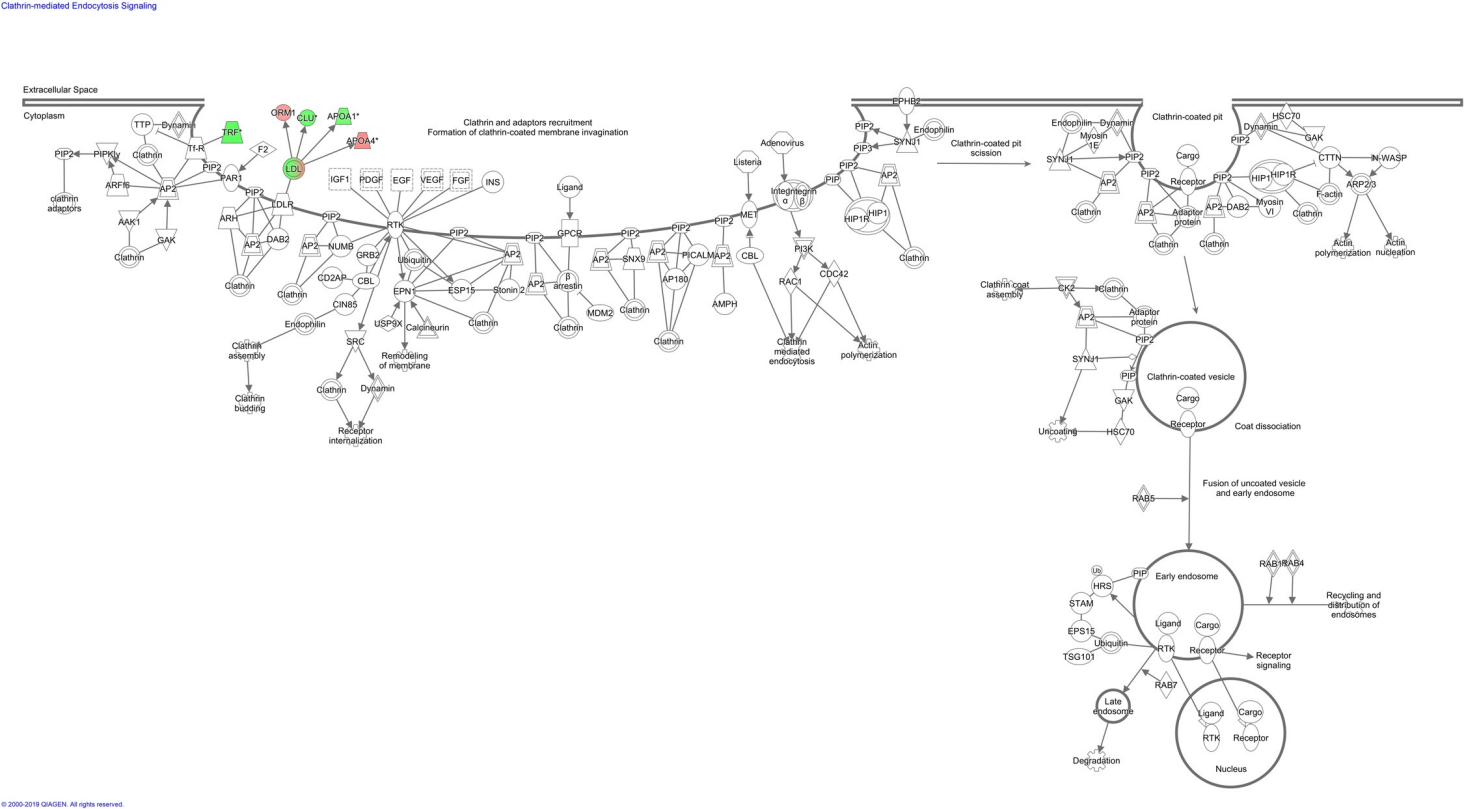
Clathrin-mediated endocytosis signalling pathway overlap with differentially expressed blood proteins between control patients and lung cancer patients before and after second cycle of chemotherapy. Red proteins are upregulated in cancer patients, green proteins are downregulated in cancer patients. White proteins were not identified in our proteomics study, but were incorporated as part of the network.

A summary of the IPA pathway analysis for blood proteins enriched in control and lung cancer patients after second cycle of chemotherapy is provided in Table 7. The top canonical pathways associated with the identified blood proteins included “FXR/RXR and LXR/RXR activation”, “acute phase response signaling” and”clathrin-mediated endocytosis signaling” (Figs 4–6 and 8), while the molecular and cellular function lists included “cell-to-cell signaling and interaction”,” lipid metabolism”, “molecular transport” and “free radical scavenging”. The physiological system development and function lists included “haematological system development and function” and “immune response”.

**Table 7:** Functional analysis (IPA) of differentially abundant proteins between the blood plasma of control patients and lung cancer patients after second cycle of chemotherapy.

**Ingenuity Canonical Pathways**	**Molecules**
LXR/RXR and FXR/RXR Activation	HPX,APOA1,APOA4,ORM1,TF,ITIH4,FGA,CLU
Acute Phase Response Signaling	HPX,HP,APOA1,ORM1,TF,ITIH4,FGA,A2M
Clathrin-mediated Endocytosis Signaling	APOA1,APOA4,ORM1,TF,CLU
**Molecular and cellular function**	**Molecules**
Free Radical Scavenging	APOA1,APOA4,GSN,HBB,HP,ITIH4,TF,CLU
Lipid Metabolism and Molecular Transport	APOA1,APOA4,CLU,GSN,A2M, HP, HBB,ORM1, AZGP1, FGA, HPX, TF
Cell-To-Cell Signaling and Interaction	A2M,APOA1,APOA4,CLU,FGA,ORM1, GSN, HBB, TF
**Physiological system development and Function**	**Molecules**
Hematological System Development and Function	A2M,APOA1,APOA4,CLU,FGA,ORM1,F13B,HP, GSN,HBB, HPX, TF, AZGP1
Immune Cell Trafficking and Organismal Development	APOA1,AZGP1,CLU,FGA,GSN,HP,HPX,TF, ORM1, HBB, LRG1,ITIH4, A2M, APOA4
Cardiovascular System Development and Function	APOA1,CLU,FGA,GSN,HP,LRG1,ORM1,TF, HPX

## Discussion

In this study, the 2D-DIGE separation was performed on depleted blood from control patients and lung cancer patients before the first and after the second cycle of chemotherapy. Analyses of gels revealed significant changes in the type and abundance of proteins and/or their proteoforms between control patients and lung cancer patients, both before and after chemotherapy. Significant changes in the type and abundance of proteins and/or their proteoforms between ADC and SCC patients were also observed.

The depletion procedure used in this study was successful, because no albumin nor immunoglobin was detected on the gels, with the exception of immunoglobin heavy chain constant α 1 membrane bound isoform 1, which is recognised as a tumour-related cell membrane protein [28]. Moreover, other proteins that tend to be removed together with albumin (called the albuminome) such as apolipoproteins, clusterin, complement inhibitor, clusterin, haptoglobin, hemopexin, leucine-rich α-2 glycoprotein, and transferrin [29] were not lost during purification, because they were identified in the depleted serum (Table 3). However, our results demonstrated clear changes in the abundance of classical or highly abundant blood plasma proteins, as defined by Strohkamp *et al*. [19]. These proteins include transferrin, 2-macroglobulin, haptoglobin, leucine-rich α-2 glycoprotein, fibrinogen, apolipoprotein A-IV, and clusterin. Given the large range (powers of 10) in the circulating concentrations of various proteins in blood plasma [29], the detection of these proteins was expected due to their high concentrations within the detection limits of 2D-DIGE.

Differences in the numbers of identified proteins between different studies employing 2D-DIGE of blood plasma proteins likely reflect differences in methodological approaches and the composition of the control and cancer patient groups. Differences in methodologies mainly concern the extraction of blood proteins for 2D-DIGE. For example, Wen *et al*. [30] only used the glycoprotein fraction of serum, which was obtained with the use of ConA affinity columns. Most studies removed high-abundance proteins from serum to varying degrees. For example, Wen *et al*. [30] removed five abundant proteins, including albumin, IgA, IgG, transferrin, and HP. Okano *et al*. [22] and Dowling et al. [31] removed six of the most abundant proteins (albumin, transferrin, haptoglobin, alpha-1-antitrypsin, IgA, and IgG). On the other hand, Rodríguez-Piñeiro et al. [32] removed 20 of the most abundant proteins (albumin, transferrin, α1-acid glycoprotein, complement C1q, IgG, fibrinogen, ceruloplasmin, complement C3, IgA, α2-macroglobulin, apolipoprotein A-1, complement C4, IgM, α1-antitrypsin, apolipoprotein A-II, plasminogen, IgD, haptoglobin, apolipoprotein B, and prealbumin). Clearly, significant differences in depletion methodology do exist and can be a significant factor in the comparative interpretation of the results of 2D-DIGE studies of the proteome of lung cancer patients. This calls for the development of standardised procedures for the preparation of serum samples for 2D-DIGE analysis.

Nevertheless, the results of our study are quite consistent with previous reports indicating the aberrant expression of plasma proteins in lung cancer [33]. To our knowledge, six studies of blood plasma from cancer patients focusing on a comparative proteomics approach using 2D-DIGE have been published, including Okano *et al*. [22,34], Dowling *et al*. [31], Hoagland *et al*. [35], Rodríguez-Piñeiro *et al*. [32] and Wen *et al*. [30]. Several proteins were identified across all studies (alpha-2-HS-glycoprotein or haptoglobin); however, proteins exclusive to a single study were also reported (alpha-2-macroglobulin isoform A and B, coagulation factor XIII, fibrinogen alpha chain and beta chain, IG heavy chain α 1 membrane bound isoform, orsomucoid 1, and zinc alpha 2 glycoprotein). It is worth mentioning that, out of the five potential plasma cancer biomarkers identified using a very different methodology (profiling of plasma proteome with monoclonal antibody libraries) [36], four proteins (alpha-1-antichymotrypsin, haptoglobin, complement C9, and leucine-rich α-2 glycoprotein precursor) were also identified using 2D-DIGE studies. Therefore, electrophoretic and immunological methods for the detection of changes in the blood plasma proteins of lung cancer patients produce similar results.

The results of our study and previous studies employing 2D-DIGE [30–32,34,35] clearly indicate that 2D-DIGE can be an effective method to identify proteins related to inflammation, especially acute phase proteins (APPs). These proteins include haptoglobin and its various forms, complement component C3, clusterin, and serum amyloid A [37–38]. Moreover, changes in other serum proteins are clearly indicated in this study and similar studies. Dowling *et al*. [37] used ELISA analysis developed specifically for five blood proteins to define abundance trends for different cancers. In this study, 2D-DIGE analysis indicated 41 proteins or their proteoforms had differential abundance (Tables 3 and 4) and it is reasonable to assume that these proteins should be targeted for future individual measurements to develop more powerful tools for cancer profiling.

It has long been recognised that there is a link between cancer and inflammation. Inflammation is known to be both a cause and a consequence of cancer [39]. Therefore, a chronic inflammatory-like state is regarded as a hallmark of cancer and is associated with cancer development and disease progression [40–41], including lung cancer [42–43]. For a long time, the potential role of APPs has been underestimated and attributed to only representing cancer epiphenomena [37]. However, recent progress in proteomics studies strongly suggests that the variable expression of APPs can be used to profile the distinct types, subtypes, and even stages of cancer. Dowling *et al*. [37] was able to indicate different abundance trends for APPs in different cancers, including lung cancer; a similar approach was successfully employed by Wang *et al*. [44]. Together, these results strongly suggest the potential of APPs for cancer fingerprinting. It is also important to note that changes in APPs in response to cancer can be clearly demonstrated despite the presence of patients with non-cancerous diseases (causing inflammatory conditions) in control groups, which was indicated in this study and in the results reported by Rodríguez-Piñeiro *et al*. [32]. This strongly supports the specificity of cancer profiling using APPs. Therefore, 2D-DIGE represents a powerful tool to explore the signature of APPs in the blood plasma of lung cancer patients.

It should be stressed that, although it is believed that APPs originate from the liver rather than from tumour cells, it is also possible that APPs can be directly produced by tumour tissue [44]. Therefore, further comparative studies are warranted to determine the origin of APPs in blood in order to better understand the relationship between APPs and cancer.

The results of our study clearly demonstrate that plasma proteins that are differentially expressed in lung cancer patients can differ in ways more nuanced than protein concentration. For example, both in this study and in that of Dowling *et al*. [31], several proteoforms of haptoglobin were identified (five in this study and four in Dowling *et al*. [31]). This suggests that changes in blood plasma proteins in response to lung cancer can be related not only to changes in their abundance, but also to post-translational modifications (PTMs). More broadly speaking, the large number of PTMs may relate to the quantitative and qualitative discrepancies between genomic, transcriptomics and their protein counterparts [45–47]. This suggestion is also supported by the recent indication of the importance of quantitative and qualitative discrepancies between genomic/transcriptomic alterations and their protein counterparts, mostly related to t. To date, the majority of the serum/plasma proteomics studies have focused on the measurement of the abundance of total proteins [48]; however, the research focus is now shifting towards studying the relationships between PTMs and cancer. These PTMs include glycosylation (including glycan-modified derivatives of haptoglobin) [35], fucosylation [49], phosphorylation, acetylation, arginine methylation, and lysine methylation [48–51], and several PTMs of histone proteins [52]. Our study provides a list of several forms of proteins that are altered in lung cancer patients, including proapolipoprotein, apolipoprotein AIV, clusterin, gelsolin, fibrinogen, haptoglobin (see above), hemopexin, transferrin, and serotransferin. Proteoforms can also contribute to mechanisms besides protein synthesis that are responsible both for the increases and decreases in protein abundance observed in this study and by other authors [30–32,34,35]. Additional studies are required to determine the exact nature of PTMs of these proteins and their usefulness for cancer profiling.

To our knowledge, we have identified, for the first time, proteins and their proteoforms that change in abundance before and after a second cycle of chemotherapeutic treatment for lung cancer. Most changes observed (4 out of 7) were in transferrin and serotransferrin. These changes likely reflect disturbances in iron turnover (iron deficiency) after chemotherapy-induced anaemia [53]. Because transferrin is an iron-binding protein, changes in its abundance in response to chemotherapy likely reflect disturbances in iron turnover. This suggestion is supported by recent findings indicating changes in transferrin levels due to chemotherapy [54–55]. The presence of transferrin proteoforms, demonstrated in this study, strongly suggests that PTMs contribute to the mechanisms of chemotherapy-induced changes in the blood plasma proteome. The identification of changes in haemoglobin abundance after chemotherapy can also be explained by chemotherapy-induced anaemia, because changes in haemoglobin abundance have also been reported [54–55]. Furthermore, changes in haemoglobin scavenger proteins, such as hemopexin, as reported in this study, are likely to be a part of the above-mentioned changes. The last protein identified in this study was complement C3 which others have documented as in the early host response to chemotherapy [56].

In this study, the number of differentially abundant proteins was higher in blood plasma obtained from lung cancer patients after a second cycle of chemotherapy compared to controls than it was in blood plasma obtained before chemotherapy compared to controls. This may be partially explained by the effects of chemotherapy itself on the blood proteome. On the other hand, the increase in several proteins likely reflects the progression of cancer development. For example, complement component 3, which can be a biomarker for chemotherapy (see above), has also been indicated as a prognostic factor for NSCLC [57–58]. For this reason, additional investigation is necessary to define the specific functions of plasma biomarkers, both for chemotherapy and cancer progression.

To our knowledge, this is the first study to directly compare the blood plasma proteome of ADC and SCC lung cancer patients using the 2D-DIGE approach [13]. Although the number of patients in this study was very restricted (four patients per cancer), our results clearly suggest that proteomics studies that profile ADC and SCC using a larger patient sample size are highly justified, because different sets of proteins can be specifically attributed to either ADC or SCC. It is especially interesting to identify vitronectin as a potential marker for SCC because this protein was identified in SCC patients before chemotherapy. This protein has been recently indicated as a potent migration-enhancing factor of cancer cells chaperoned by fibrinogen [59]. Therefore, additional studies are warranted to test the usefulness of vitronectin as a potential marker of SCC.

Our results clearly indicate the heterogeneity in profiles of particular proteins or their proteoforms in individual patients. This agrees with established knowledge that the performance of individual markers is variable due to their low sensitivity and specificity [1]. Therefore, it is unlikely that a single biomarker for any particular form of cancer can be identified; rather, a multi-marker approach is recommended [10–11].

In summary, our study has extended the list of potential lung cancer biomarkers. Our results emphasise the potential role of inflammatory proteins as biomarkers of lung cancer. Chemotherapy is accompanied by changes in proteins involved in anaemia. SCC can be distinguished from ADC using proteomics profiling, with a special emphasis on vitronectin. The presence of numerous proteoforms for several biomarkers warrants an investigation of the relationship between PTMs and cancer.

## Supporting information

S1 Table The comparison of selected protein spots (196, 374, 383, 588, 1014, 1046, 1252, 1263) across gels with serum samples from patients with a lung cancer at stage I and III. The serum sample from patient with a lung cancer at stage I is marked in pink (gel no. 55259 Cy 3).
(DOCX)

S2 Table The comparison of selected protein spots (493, 887, 1019, 1572) across gels with blood plasma from lung cancer patients before first cycle of chemotherapy and after second cycle of chemotherapy. (DOCX)

S1 Fig Immunoblotting validation of transferrin, fibrinogen α chain and vitronectin of control and lung cancer serum samples before and after second cycle of chemotherapy (A). The images of the gels after SDS-PAGE electrophoresis (B) and membranes after transfer and before incubation with primary antibodies (C) are provided to check the quality of the serum samples separations and as a control for equal protein loading among samples. Black frames indicate which parts of the blots were presented as Fig 3.
(DOCX)
