# Supplementary material for: Identification of protein changes in the blood plasma of lung cancer patients subjected to chemotherapy using a 2D-DIGE approach
Source: PLoS One. 2019 Oct 17;14(10):e0223840. doi: 10.1371/journal.pone.0223840 (PMC6797170; doi:10.1371/journal.pone.0223840)
Supplement: S1 Table — The serum sample from patient with a lung cancer at stage I is marked in pink (gel no. 55259 Cy 3). (DOCX) [file pone.0223840.s001.docx]

**Supplementary Table 1.** The comparison of selected protein spots (196, 374, 383, 588, 1014, 1046, 1252, 1263) across gels with serum samples from patients with a lung cancer at stage I and III. The serum sample from patient with a lung cancer at stage I is marked in pink (gel no. 55259 Cy 3).

| **Spot no.** | **Gels** | **Graph view** |
| --- | --- | --- |
| **196** | 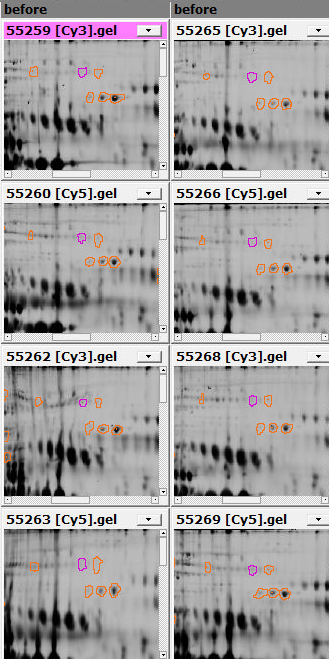 | 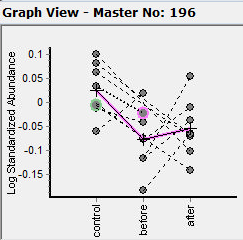 |
| **374** | 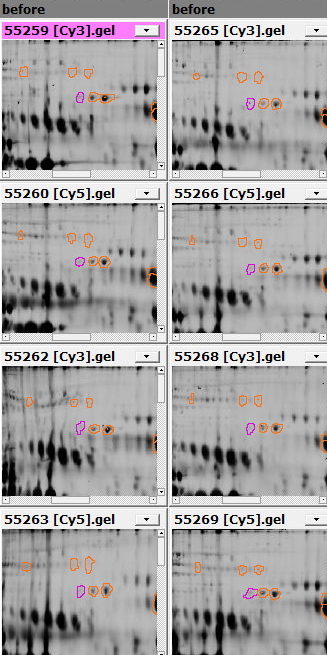 | 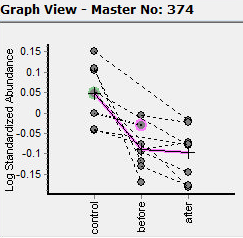 |
| **383** | 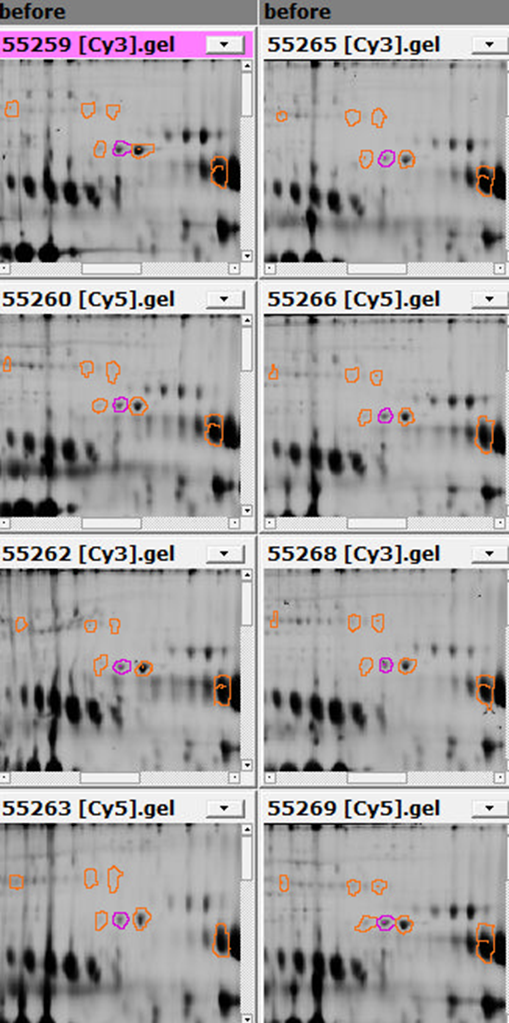 | 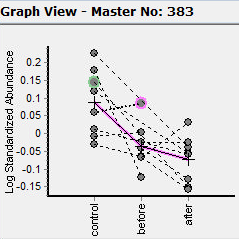 |
| **588** | 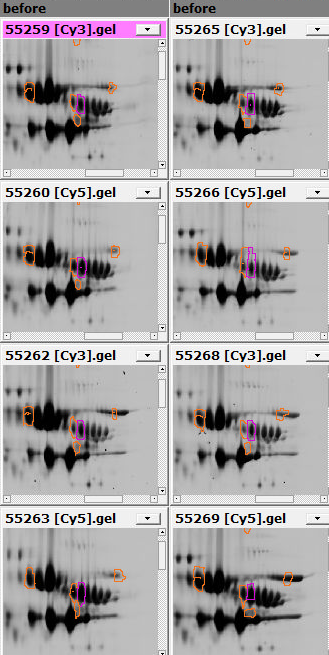 | 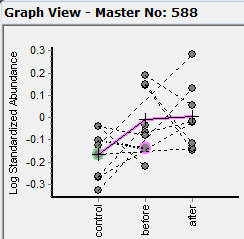 |
| **1014** | 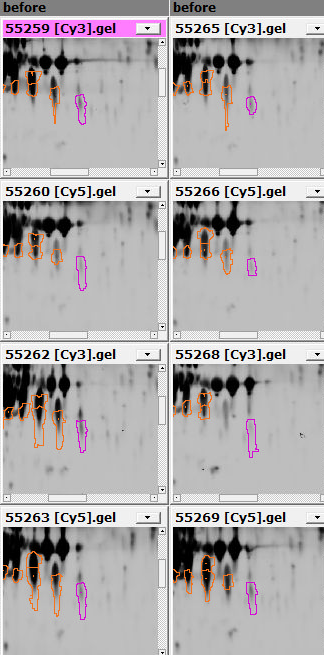 | 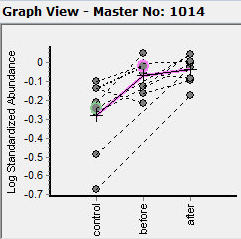 |
| **1046** | 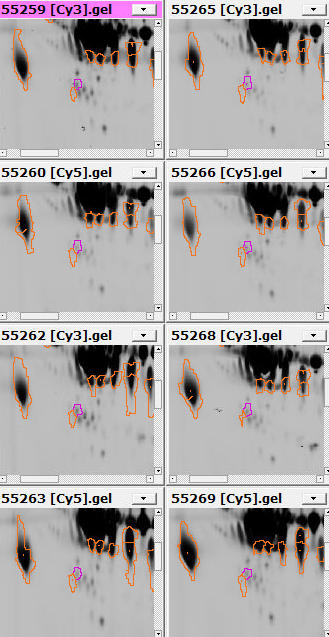 | 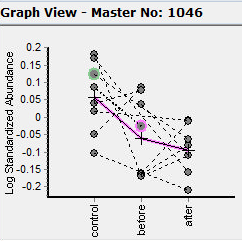 |
| **1252** | 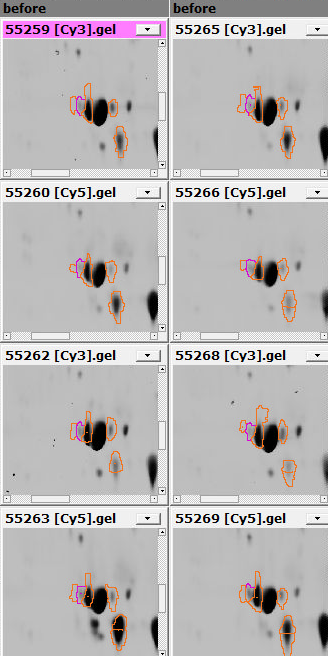 | 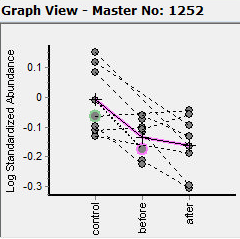 |
| **1263** | 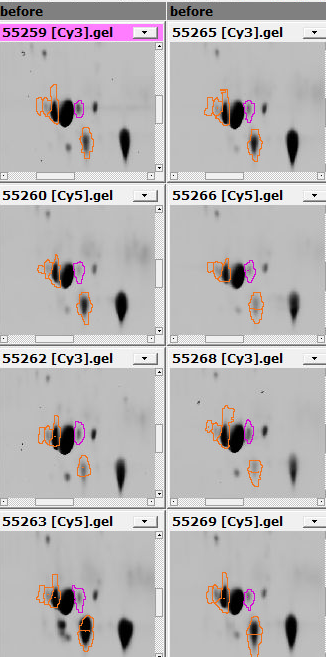 | 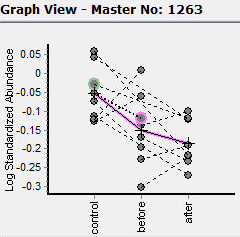 |

The serum sample from patient with a lung cancer at stage I is marked in pink (gel no. 55259 Cy 3).

control - blood plasma from control patients,

before – blood plasma from lung cancer patients before first cycle of chemotherapy,

after – blood plasma from lung cancer patients after second cycle of chemotherapy.
