# Supplementary material for: Identification of protein changes in the blood plasma of lung cancer patients subjected to chemotherapy using a 2D-DIGE approach
Source: PLoS One. 2019 Oct 17;14(10):e0223840. doi: 10.1371/journal.pone.0223840 (PMC6797170; doi:10.1371/journal.pone.0223840)
Supplement: S2 Table — (DOCX) [file pone.0223840.s002.docx]

**Supplementary Table 2.** The comparison of selected protein spots (493, 887, 1019, 1572) across gels with blood plasma from lung cancer patients before first cycle of chemotherapy and after second cycle of chemotherapy.

| **Spot no.** |  | **Graph view** |
| --- | --- | --- |
| **493** | 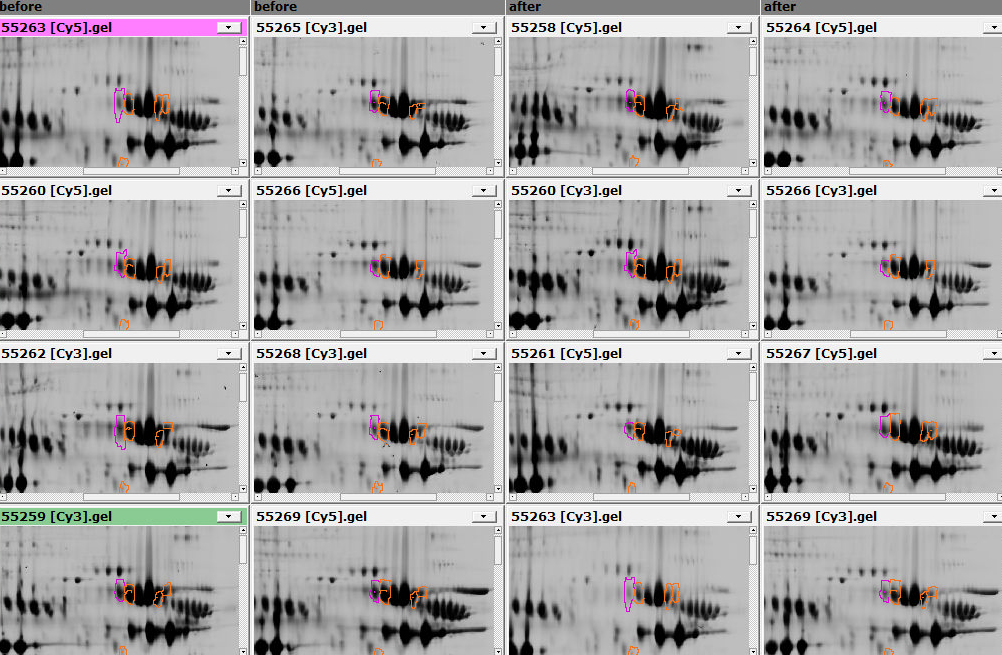 |  |
| **887** | 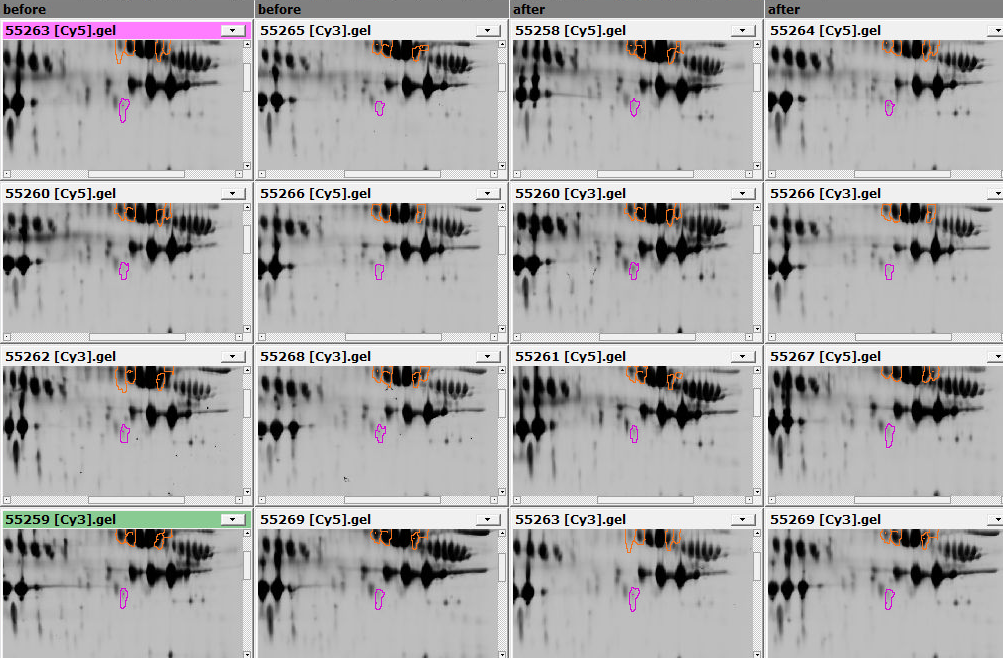 |  |
| **1019** | 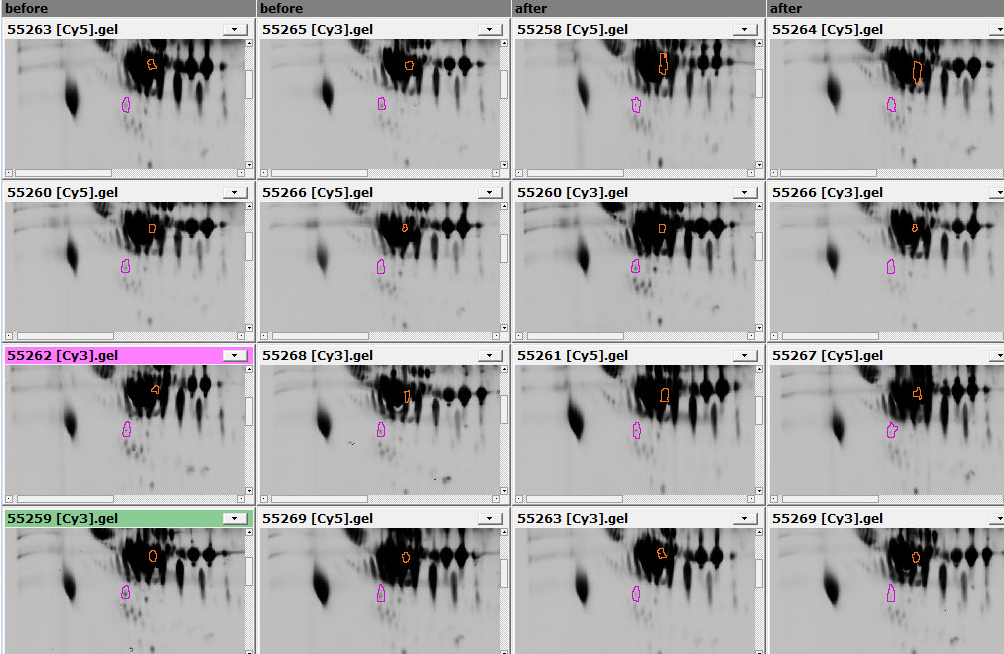 |  |
| **1572** | 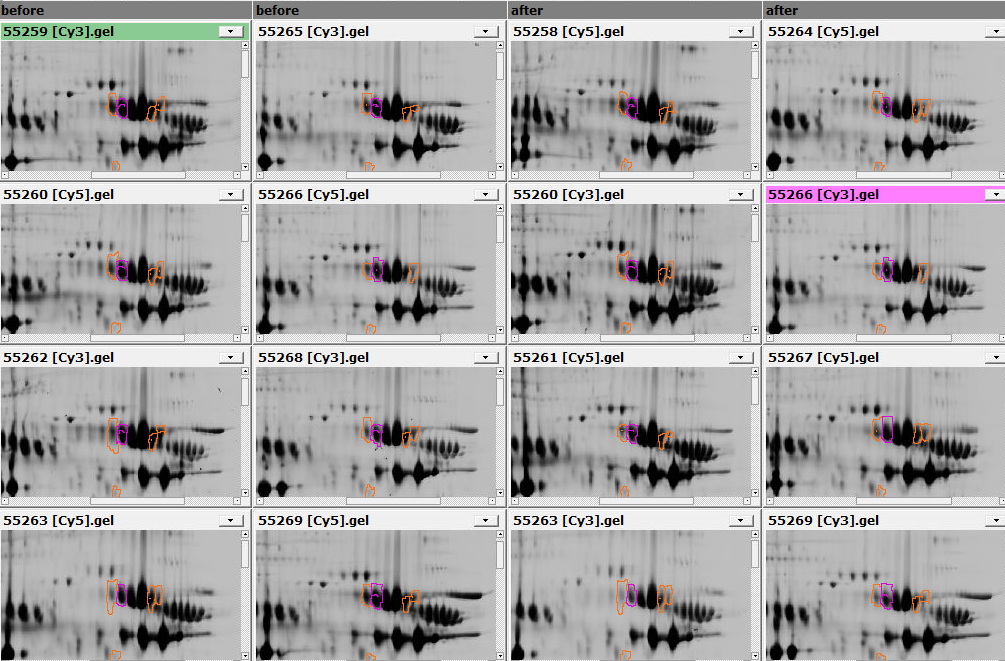 |  |

before – blood plasma from lung cancer patients before first cycle of chemotherapy,

after – blood plasma from lung cancer patients after second cycle of chemotherapy.

Gels in red boxes indicate outliers
