# Supplementary material for: Identification of protein changes in the blood plasma of lung cancer patients subjected to chemotherapy using a 2D-DIGE approach
Source: PLoS One. 2019 Oct 17;14(10):e0223840. doi: 10.1371/journal.pone.0223840 (PMC6797170; doi:10.1371/journal.pone.0223840)
Supplement: S1 Fig — Immunoblotting validation of transferrin, fibrinogen α chain and vitronectin of control and lung cancer serum samples before and after second cycle of chemotherapy (A). The images of the gels after SDS-PAGE electrophoresis (B) and membranes after transfer and before incubation with primary antibodies (C) are provided to check the quality of the serum samples separations and as a control for equal protein loading among samples. Black frames indicate which parts of the blots were presented as Fig 3. (DOCX) [file pone.0223840.s003.docx]

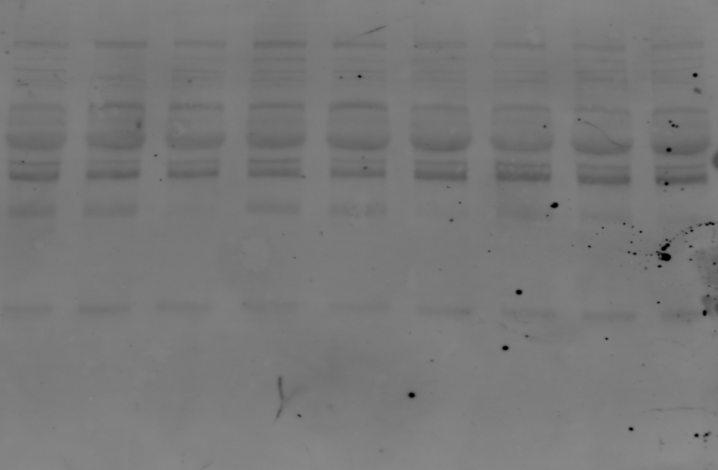

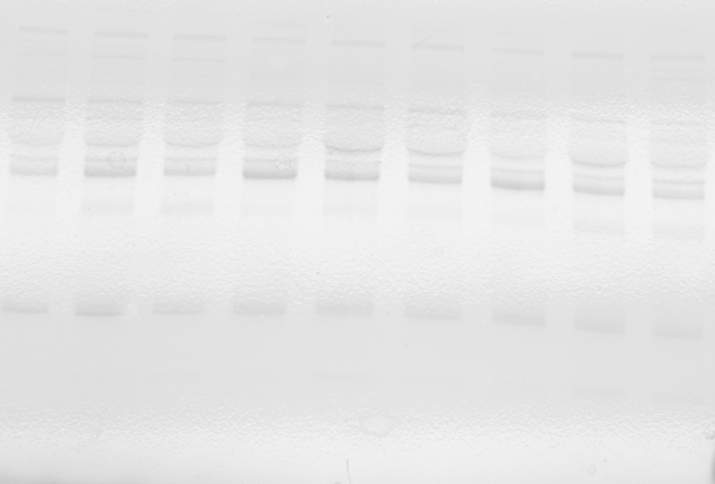


202

114

83

46

33

27

17


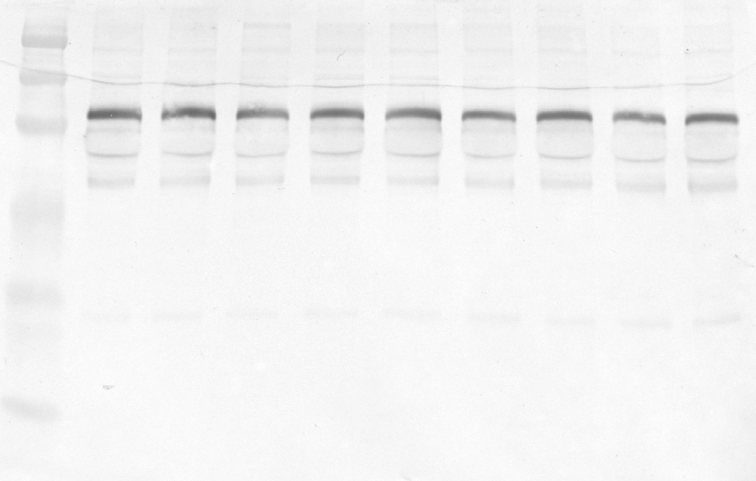


**Control Before After Control Before After Control Before After**

**transferrin**

**B**

**Control Before After Control Before After Control Before After**

**Control Before After Control Before After Control Before After**

**A**

**C**

**Control before after**


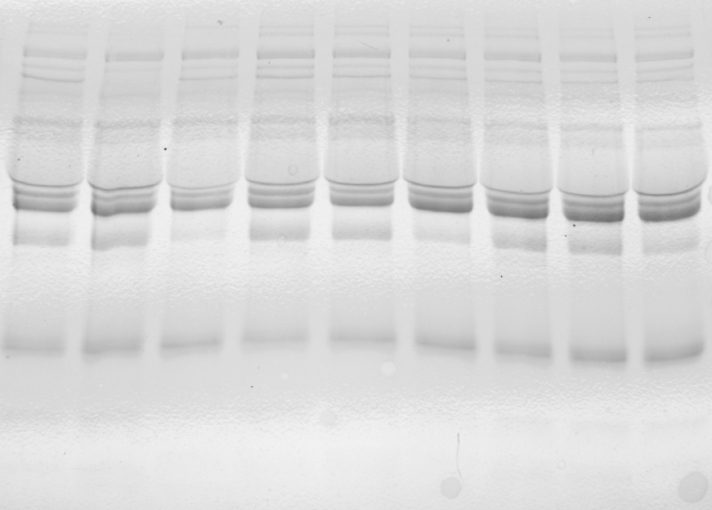


202

114

83

46

33

27

17


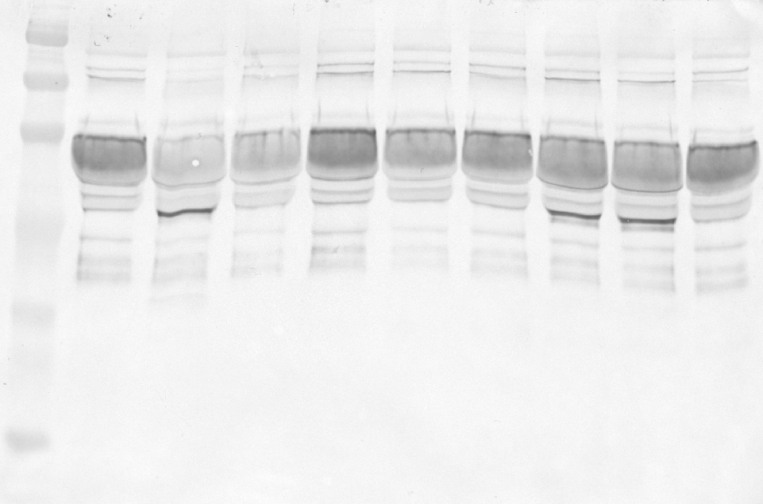


**After Before Control After Before Control Control Before After**

**fibrinogen**

**After Before Control After Before Control Control Before After**

**After Before Control After Before Control Control Before After**


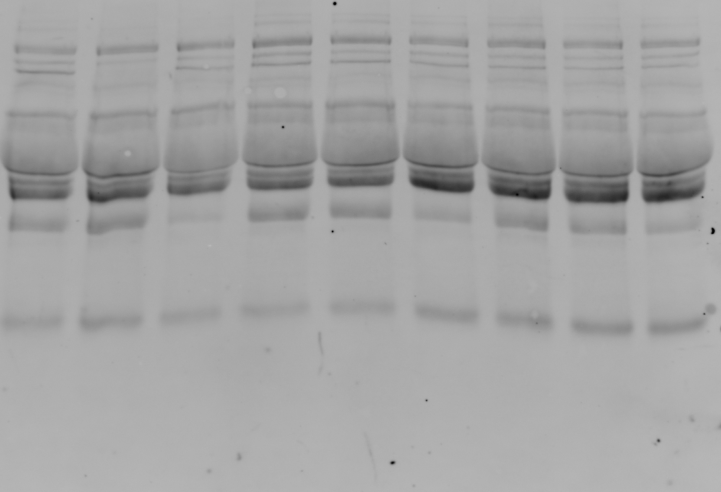


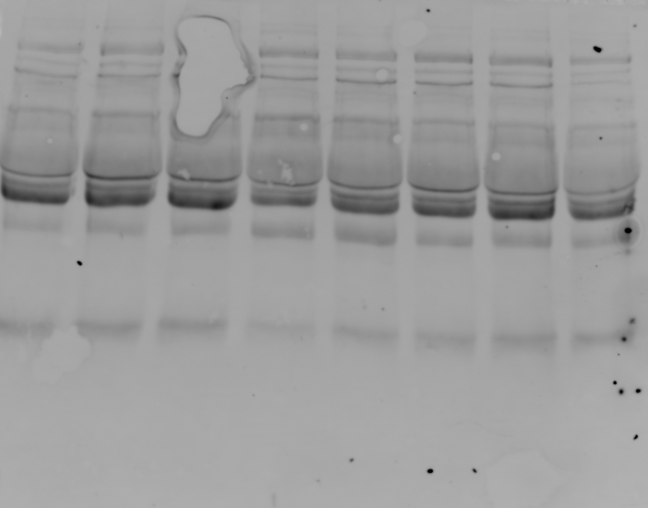

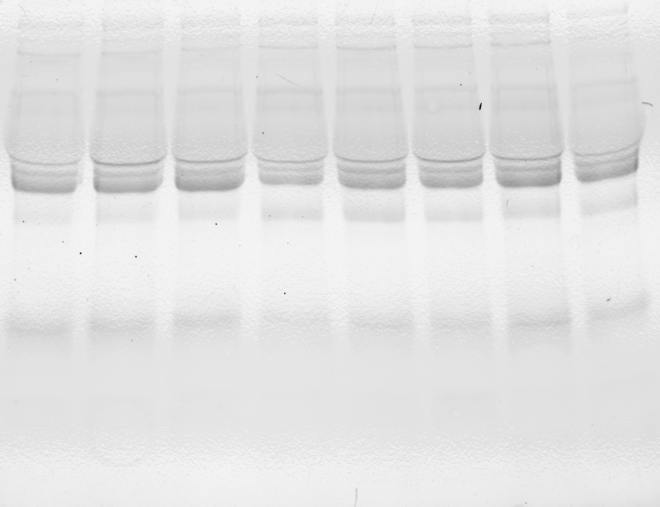


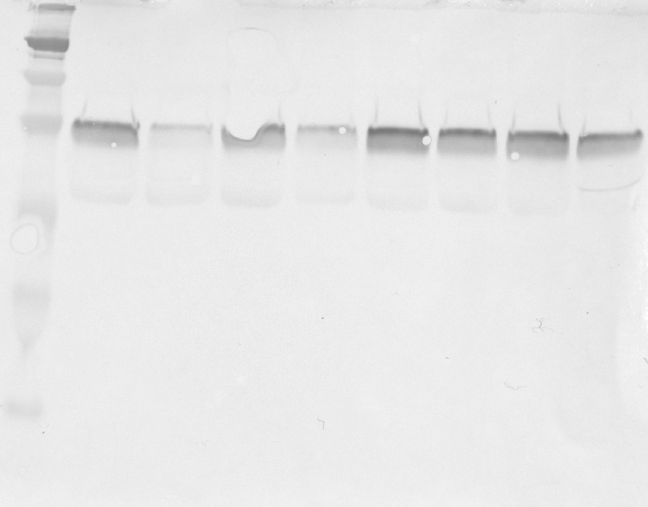


ADC SCC

202

114

83

46

33

17

**vitronectin**

ADC SCC

ADC SCC

Supplementary Fig. 1. Immunoblotting validation of transferrin, fibrinogen α chain and vitronectin of control and lung cancer serum samples before and after second cycle of chemotherapy (A). The images of the gels after SDS-PAGE electrophoresis (B) and membranes after transfer and before incubation with primary antibodies (C) are provided to check the quality of the serum samples separations and as a control for equal protein loading among samples. Black frames indicate which parts of the blots were presented as Fig. 3
